# Velocity-weakening and -strengthening friction at single and multiasperity contacts with calcite single crystals

**DOI:** 10.1073/pnas.2112505119

**Published:** 2022-05-25

**Authors:** Binxin Fu, Rosa M. Espinosa-Marzal

**Affiliations:** ^a^Department of Civil and Environmental Engineering, University of Illinois at Urbana–Champaign, Urbana, IL 61801;; ^b^Department of Materials Science and Engineering, University of Illinois at Urbana–Champaign, Urbana, IL 61801

**Keywords:** friction, calcite, contact aging, rate-and-state friction law, atomic force microscopy

## Abstract

Although earthquakes are one of the most notorious natural disasters, a full understanding of the underlying mechanisms is still lacking. Here, nanoscale friction measurements were performed by atomic force microscopy (AFM) on calcite single crystals with an oxidized silicon tip to investigate the influence of roughness, contact aging, and dry vs. aqueous environment. In dry environments, smooth and rough calcite surfaces yielding single- and multiasperity contacts, respectively, exhibit velocity-weakening ($\beta_{D}\ln V$) or neutral friction at slow sliding velocities and velocity-strengthening friction ($\alpha_{D}\ln V$) at higher velocities, while the transition shifts to slower velocities with an increase in roughness. The origin of the velocity-weakening friction is determined to be contact aging resulting from atomic attrition of the crystalline surface. Friction measurements in aqueous environment show evidence of pressure solution at sufficiently slow sliding velocities, which not only significantly reduces friction on single-and multiasperity contacts but also, eliminates atomic attrition and thereby, velocity-weakening friction. Importantly, the friction scaling law evolves from logarithmic ($\beta_{D}\ln V$) into linear ($\alpha_{PS}V$), deviating from commonly accepted rate-and-state friction (RSF) laws; this behavior extends over a wider range of velocities with higher roughness. Above a transition velocity, the scaling law remains logarithmic ($\alpha_{W}\ln V$). The friction rate parameters $\alpha_{D}$, $\beta_{D}$, $\alpha_{PS}$, and $\alpha_{W}$ decrease with load and depend on roughness in a nonmonotonic fashion, like the adhesion, suggesting the relevance of the contact area. The results also reveal that parameters and memory distance differ in dry and aqueous environments, with implications for the understanding of mechanisms underlying RSF laws and fault stability.

## Significance

The empirical nature of rate-and-state friction (RSF) equations remains a drawback to their application to predict earthquakes. From nanoscale friction measurements on smooth and rough calcite crystals, a set of parameters is analyzed to elucidate microscopic processes dictating RSF. We infer the influence of roughness on the velocity dependence of friction in dry environment and that atomic attrition leads to stick–slip instabilities at slow velocities. In fault dynamics, stick–slip is associated with seismic slips. The aqueous environment eliminates atomic attrition and stick–slip and dissolves calcite under pressure. This yields remarkable lubrication, even more so in rough contacts, and suggests an alternative pathway for seismic slips. This work has implications for understanding mechanisms dictating fault strength and seismicity.

Rate-and-state friction (RSF) constitutive equations (1–3) have been commonly used to describe the friction and slip behavior of rocks and fault gouge. This model describes the kinetic friction coefficient μ as the sum of a rate term, which increases logarithmically with the sliding velocity (V), and a state term, which depends on a time-dependent state variable (θ), and is understood as the average lifetime of frictional contacts that are replaced by new contacts after sliding a distance $D_{c}$, the memory distance:

[1] $$\mu =\mu_{0}+a\cdot \ln V/V_{0}+b\cdot \ln(\theta \cdot V_{0}/D_{c}),$$

with a and b being empiric material parameters; the subindex “0” represents a reference state. The direct effect (the second term) represents a thermally activated sliding process (i.e., the thermal energy assists the slip process more so at slow sliding velocities, which leads to an increase of friction with velocity). The third term represents the evolution (indirect) effect, whereby the state variable θ evolves with contact time. In steady state, the state variable can be given as $\theta_{ss}=D_{c}/V$, which yields the following expression for the kinetic friction coefficient (4):

[2] $$\mu_{ss}=\mu_{0}+\left(a-b\right)\cdot \ln V/V_{0}.$$

Hence, the steady-state kinetic friction coefficient can either decrease or increase with velocity depending on the sign of a−b, which classifies fault materials as either velocity-weakening (a−b<0) or velocity-strengthening materials (a−b>0).

RSF laws are often applied to predict fault stability. If the stiffness of velocity-weakening materials is smaller than a critical value $k_{\mathrm{crit}}=\sigma_{n}(b-a)/D_{c}$ for a given normal stress $\sigma_{n}$, a decrease of friction with the slip velocity will yield an instability (or stick–slip) (3). Such instabilities are generally associated with seismic slips (5). Regarding the effect of water, RSF equations predict that fluid overpressure ($P_{f}$) decreases the effective stress ($\sigma_{n}-P_{f}$) and thereby, $k_{\mathrm{crit}}$, and therefore, water should promote aseismic creep. In contrast to predictions based on RSF equations, seismologic studies (6) as well as laboratory experiments (7) suggest that high fluid pore pressure can trigger dynamic slip instability, despite the velocity-strengthening frictional response of the materials. The origin of this behavior is the subject of current studies.

Nanoscale friction measurements can help us understand the mechanisms underlying the frictional response of geological materials (8–17). The kinetic friction at nanoscale single-asperity dry contacts exhibits often increasing and/or decreasing trends with ln(V), like the direct and evolution effects in the RSF law. The evolution effects are also reflected in an increase of static friction with contact time, called contact aging (8). Contact aging is typically associated with an increase in contact area due to creep (called “contact quantity”). Several processes have been determined to contribute to increasing the contact area in the context of multiasperity contacts: enlargement of individual contacts, formation of new contacts, and merging of neighboring contacts (18). Recent studies have proposed that contact aging at silica–silica contacts can also result from chemical bonding (so-called “contact quality”) (9) and have related the memory distance to the average sliding distance before an activated reaction site becomes passivated (10). A few atomic force microscopy (AFM) studies (14–17) have reported the stress-induced dissolution of minerals also while sliding. Our nanoscale studies with an AFM tip sliding on atomically smooth calcite crystals (11, 12) showed a remarkable decrease of the kinetic friction coefficient and a prominent deviation from lnV at high normal stresses. Experiments with a surface forces apparatus (SFA) revealed that the decrease in friction happens concurrently with the pressure-induced dissolution of calcite (13), a lubrication mechanism that we called “pressure solution–facilitated slip” (11).

In a further attempt to bridge the gap between nanoscale friction and RSF laws and fault dynamics, we study here the effect of nanoscale roughness on the friction between a thermally annealed silicon tip (190 nm in radius) and calcite single crystals both in a dry environment and in equilibrium with water. Single crystals of calcite, the simplest crystalline polymorph of calcium carbonate and a very abundant mineral in the lithosphere, were cleaved along the ($10\bar{1}4$) plane to prepare atomically flat surfaces for single-asperity friction force measurements (19). For multiasperity friction force measurements, rough calcite surfaces were prepared by polishing the ($10\bar{1}4$) plane of calcite. We found a nonmonotonic variation of friction with velocity on smooth calcite surfaces in a dry environment (first decreasing and then increasing with the logarithm of velocity), while the velocity-weakening friction became less prominent at multiasperity contacts. Upon the addition of water, the nonmonotonic frictional response was greatly eliminated. Here, a linear change of friction with velocity was measured at sufficiently slow sliding velocities on smooth and rough surfaces concurrent with the pressure-induced dissolution of calcite, which transitioned into a logarithmic function of the velocity at high sliding velocities. We discuss our results in the context of single- vs. multiasperity contacts, contact aging, atomic attrition, and pressure solution, and we relate these mechanisms to macroscopic RSF laws and field observations.

## Results and Discussion

### Adhesion, Contact Area, and Stress.

**Fig. 1.**
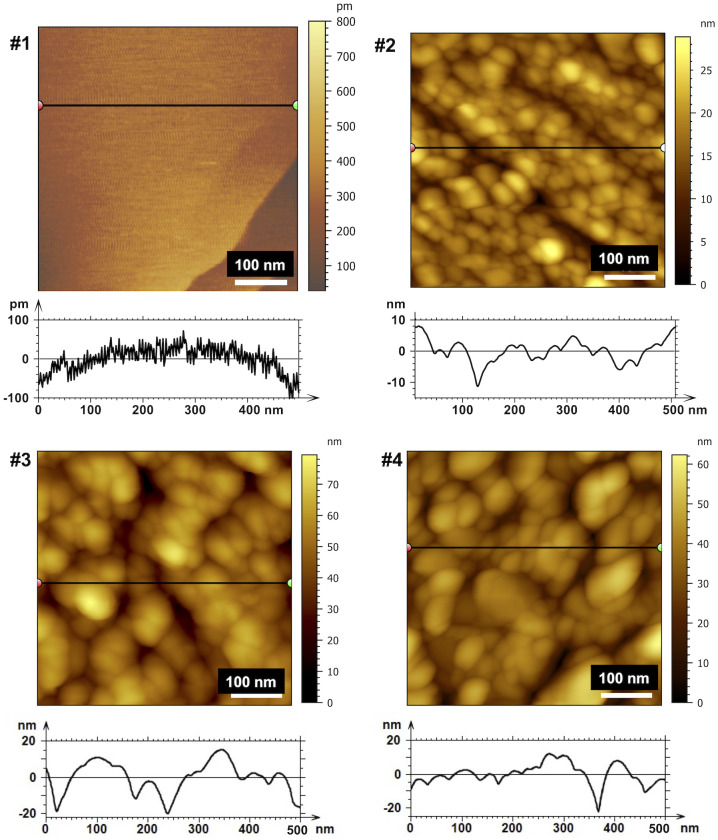
Representative AFM topographical images (500 nm × 500 nm) and corresponding cross-sections of calcite surfaces #1, #2, #3, and #4. AFM images were taken in air in tapping mode with a sharp tip. The asperity size significantly increased from #1 to #4, as indicated by the profiles (note the different *y* axes). Representative 6-µm × 6-µm and 1-µm × 1-µm images of calcite surfaces are shown in *SI Appendix*, Figs. S1 and S2.

Pieces of calcite cleaved along the ($10\bar{1}4$) plane were polished using diamond paper with different grit sizes, and images of the calcite surfaces were obtained at several magnifications (*Materials and Methods*). Representative images (500 nm × 500 nm) are shown in Fig. 1; 1-µm × 1-µm and 6-µm × 6-µm images are shown in *SI Appendix*, Figs. S1 and S2, respectively. The Mountains9 software was used to analyze the surface roughness, and the parameters are summarized in Table 1. Because the contact with the blunt tip is mainly dictated by the distribution of the small asperities and these are not captured in the 6-µm × 6-µm images, we chose the smaller images for the analysis. The rms roughness $R_{q}$ values of the polished calcite crystals are 5.0 ± 0.1, 9.9 ± 1.4, and 14.6 ± 3.9 nm for #2, #3, and #4, respectively, while the average distances between asperities $\lambda_{1}$ are 18.4 ± 1.4, 38.0 ± 6.9, and 21.6 ± 0.4 nm for #2, #3, and #4, respectively. Surface #4 has a second characteristic peak-to-peak distance with $\lambda_{2}$ = 165 ± 19 nm. The number density of asperity peaks is thus largest for #2 followed by #4 and #3. The arithmetic mean peak radius is also shown in Table 1 as well as the maximum and average peak height. The rms roughness of the cleaved calcite (Fig. 1, #1) is orders of magnitude smaller (0.101 ± 0.047 nm), but the atomic steps are expected to affect friction force measurements since the sliding distance (6 µm) is much larger than the distance between the steps. In the following, the calcite surfaces are labeled as “smooth” or #1, 5-nm rms or #2, 10-nm rms or #3, and 15-nm rms or #4. It is to be noted that several calcite single crystals were used for friction force measurement in air and aqueous environment. While we only show representative images in Fig. 1 and *SI Appendix*, Figs. S1 and S2, Table 1 shows the average results for the prepared surfaces.

**Table 1.** Topographical characteristics

	0.5- × 0.5-µm rms (nm)	1- × 1-µm rms (nm)	*r* (nm)	λ_1_ (nm)	*h_max_* (nm)	Mean *h* (nm)
#1	0.1 ± 0.04	0.101 ± 0.047			0.28 ± 0.04	0.21 ± 0.02
#2	5.0 ± 0.1	4.1 ± 0.84	23.3 ± 4.9	18.4 ± 1.4	16.6 ± 4	17.5 ± 1
#3	9.9 ± 1.4	8.0 ± 1.0	24.6 ± 0.9	38.0 ± 6.9	29.9 ± 2	39.8 ± 11
#4	14.6 ± 3.9	16.3 ± 5.7	29 ± 3	21.6 ± 0.4	42 ± 10	36.4 ± 12

Average values for rms roughness, peak radius, lateral interparticle distance, maximum peak height, and mean peak height were obtained on at least three images per surface area and roughness. The values shown were obtained on images of 1,000 nm × 1,000 nm.

**Fig. 2.**
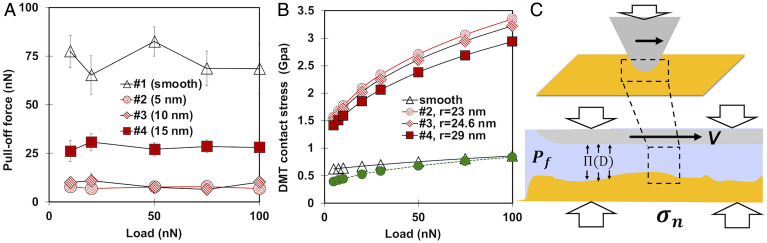
(*A*) Pull-off force on smooth and roughened calcite single crystals measured with a blunt Si tip (190-nm radius) as a function of normal load. The rms roughness of each surface is given. The error bar gives the standard variation of the pull-off force at each load. (*B*) Average contact stress as a function of normal load estimated via the DMT model on smooth surfaces and on single asperities with radii of 23, 24.6, and 29 nm. The green dashed line assumes that the load is carried out by four asperities of 23 nm in radius. Note that the asperities are assumed to be smooth, and hence, this is a simplified geometry. (*C*) Conceptual picture of the lubricated contact between the tip and calcite, illustrating the balance between the normal stress, fluid pressure, and disjoining pressure *П* (*D*). This balance is assumed to be maintained during sliding with concurrent dissolution of calcite.

The pull-off force measured with a thermally annealed tip (*SI Appendix*, Fig. S3) on smooth and rough surfaces as a function of normal load in dry nitrogen is shown in Fig. 2*A*. The largest values of the pull-off force are obtained on smooth calcite (#1) as expected (20, 21). The pull-off force changes nonmonotonically with increasing rms roughness, first decreasing significantly from ∼72.5 ± 7.2 nN on surface #1 to ∼7.8 ± 0.4 nN on surface #2 and ∼8.7 ± 1.6 nN on #3 and then, increasing to ∼28.1 ± 1.8 nN on surface #4.

While polishing can lead to amorphization of the surface layer or Beilby layer of calcite (22), it is well accepted that after equilibration, this layer recrystallizes (23). Although the Beilby layer of calcite becomes crystalline, the surface properties are altered, and Beilby (24) found that the surface is harder than the original undisturbed face of the crystal. The influence of the hardness variation on the tip–surface interaction (i.e., the pull-off force) is expected, however, to be much smaller than the effect of surface topography. In fact, the nonmonotonic change of the pull-off force with rms roughness in multiasperity contacts can be justified through the contribution of contact interactions and noncontact interactions with the surface underneath the asperities based on Rumpf and Rabinovich models, among others (20, 21). Because the peak-to-peak distance is largest for surface #3 (Table 1) and hence, fewer asperities are within the contact in this case compared with #4 and #2, it is expected that the contact area of this surface with the tip is the smallest. A precise estimation of the contact area is, however, not possible since noncontact interactions become relevant in this range of roughness, and the tip can come in contact with valleys between the asperities, which also contributes to the increase of the contact area (25).

Fig. 2*A* shows that the adhesion force is independent of the load, which indicates that the enlargement of the contact area with load as a result of plastic or elastoplastic deformation is negligible (26). This can be due to the existence of multiasperity contacts, which reduces the contact stress. To estimate the failure of the contact under compressive stress, the selected criterion corresponds to the condition at which the maximum compressive stress is equal to the yield stress of the tip or calcite, whichever is smaller. Fig. 2*B* shows the contact stress as a function of applied load on smooth calcite as well as on single asperities of different radii, both estimated using the Derjaguin–Muller–Toporov (DMT) model (*Materials and Methods*). Note that this approach does not provide a precise stress distribution; first, it calculates the stress supported by only one asperity, despite the multiasperity nature of the contact, and second, it neglects the undetectable roughness by our AFM. The arithmetic mean of the asperity radius r was used to estimate the average contact stress. The failure stress of silicon at compression varies between 5.0 and 9.0 GPa (27). The silicon surface exposed to air for a long time is covered by a layer of native oxide (SiOx), with a thickness of ∼1 to 2 nm or even less. During thermal annealing of silicon tips in air, oxidation of the surface may be enhanced, and this layer can be thicker (28). A clear determination of the surface chemistry is, however, not possible because this layer can be worn out during friction measurements and reform as soon as the tip is in contact with air. The yield stress of silica is ∼5.43 GPa (29), and the maximum estimated value of the contact stress is ∼3.5 GPa on a single asperity; hence, it is unlikely that the tip fails during friction measurements. This is supported by the small change of the tip radius over the course of the friction measurements (*SI Appendix*, Fig. S3). On the other hand, the yield stress of calcite at room temperature has been reported to be in the range from 0.5 to 1.84 GPa depending on the model used to analyze the results from micropillar compression and nanoindentation experiments (30). Hence, failure could happen if only one single asperity would support the load. Because more than one asperity is in contact with the tip, the stress should significantly decrease, thereby hindering failure; an example is in Fig. 2*B* (green markers). We recognize that this is only a rough estimation, but it is consistent with our pull-off force measurements (Fig. 2*A*).

### Friction in Dry Environment.

**Fig. 3.**
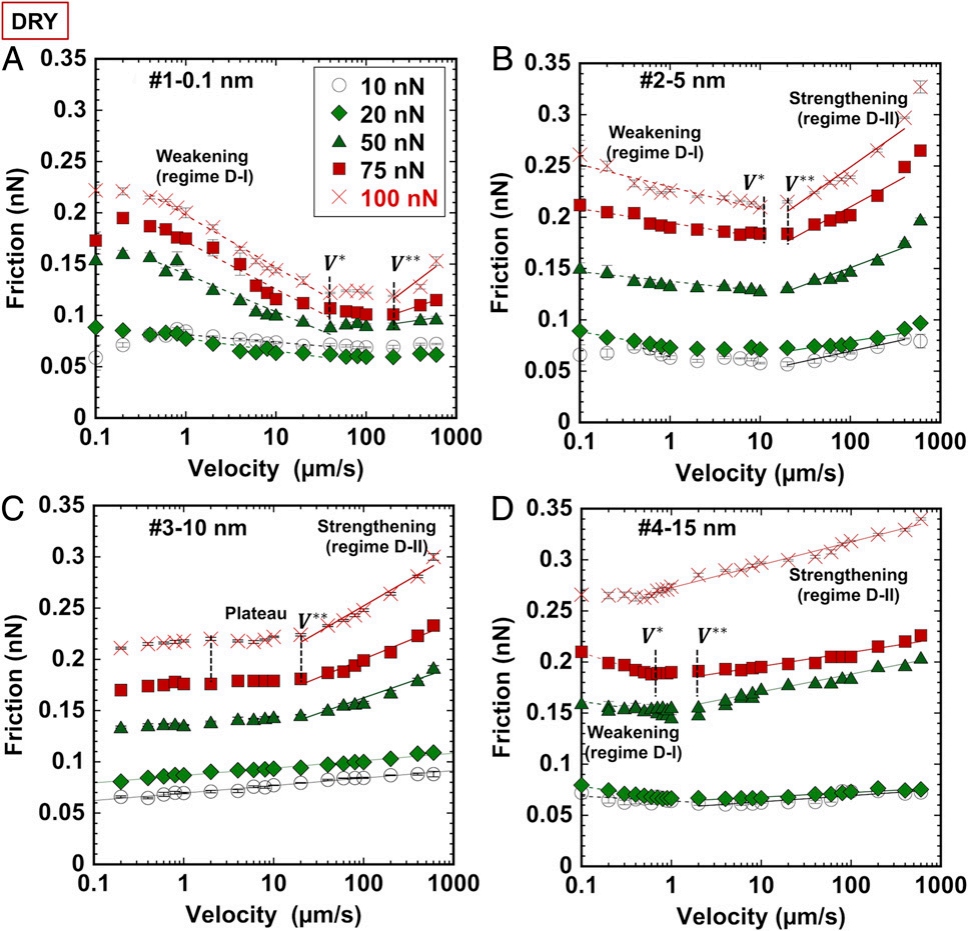
Friction force between a blunt Si tip and calcite with rms roughness values of (*A*) 0.1 nm (#1; smooth), (*B*) 5 nm (#2), (*C*) 10 nm (#3), and (*D*) 15 nm (#4) as a function of sliding velocity and normal load in dry N_2_ to avoid water contamination. The marker legend used in this paper is as follows: empty circles for 10 nN (black), diamonds for 20 nN (green), triangles for 50 nN (dark green), squares for 75 nN (red), and crosses for 100 nN (red). The error bars give the variation in friction over eight friction loops at each speed. The lines illustrate the logarithmic change of friction during velocity weakening (regime D-I; dashed lines) and velocity strengthening (regime D-II; full lines). The plateau between these $V^{*}$ and $V^{**}$ is significant. Note that the *x* axis is on a logarithmic scale and that the *y* axis is on a linear scale. The lines indicate logarithmic trends and are to guide the eye. The quantitative agreement between replica experiments is outstanding except for surface #4, which sometimes showed a less pronounced velocity-weakening regime, likely depending on the local topography. The fits of the models to the experimental results are in *SI Appendix*, Fig. S6, and the fitting parameters are in *SI Appendix*, Table S1.

Fig. 3 shows representative results of the friction force ($F_{L}$) between an AFM tip and smooth and rough calcite surfaces as a function of sliding velocity (V) and normal loads (L) between 10 and 100 nN. The cell is continuously purged with nitrogen, and hence, we expect the relative humidity to remain low during the duration of the experiment. Good agreement was found with friction measurements carried out with different AFM tips (*cf* Fig. 3 and *SI Appendix*, Fig. S4). The relation between friction and load is shown in *SI Appendix*, Fig. S5 and is consistent with the existence of a single-asperity contact between tip and smooth surface and multiasperity contact between the tip and the rough calcite surfaces. On smooth calcite (Fig. 3*A*, #1), the friction force exhibits a prominent decreasing trend with an increase in velocity (i.e., a velocity-weakening friction [regime D-I] and a less prominent velocity-strengthening friction at higher velocities [regime D-II]). We identify two transition velocities $V^{*}$ and $V^{**}$, which define the end and the start of the velocity-weakening and velocity-strengthening regimes, respectively. For smooth calcite (#1), $V^{*}$ is ∼40 µm/s, while friction increases with velocity above $V^{**}$ ∼ 200 µm/s. Only a few data points were measured above $V^{**}$, and hence, the relation between friction and velocity is uncertain; however, we describe it via a logarithmic relation $F_{L\;}∼\alpha_{D}\ln V$, as it is common for friction at single-asperity (nanoscale) contacts based on the thermally activated Prandtl–Tomlinson model (31). $\beta_{D}$ is the friction rate parameter of the logarithmic decrease (i.e., $F_{L\;}∼\beta_{D}\ln V$ at *V* < $V^{*}$). Note that $\alpha_{D}$ and $\beta_{D}$ are defined as a−b in Eq. **2**; hence, $\alpha_{D}$ is positive, and $\beta_{D}$ is negative.

For multiasperity contacts, the mechanokinetic model has successfully reproduced friction measurements between other ionic atomically smooth crystals (NaCl) and Si tips (32). The mechanokinetic model considers that the formation and rupturing processes of multiple atomic contacts are thermally activated, and the interplay between them may lead to a complex dependence of friction on tip velocity and sample temperature. Experiments and models showed a velocity-weakening regime followed by a weak increase in friction with velocity for smooth NaCl crystals (32), like in Fig. 3*A*. They also showed a prominent increase in friction with velocity prior to the velocity-weakening regime but at slower velocities than those investigated here; yet, the presence of either a peak or an increase of friction with velocity is evident at some loads and slow velocities (arrows in *SI Appendix*, Fig. S6) (surface #1, *V* ∼ 0.1 to 0.2 µm/s), consistent with this previous work. This peak is also reminiscent of the frictional behavior of calcite gouge, which shows a strong velocity-strengthening frictional behavior at low velocities that then evolves toward velocity-weakening friction behavior at higher velocities (33). Instrument limitations do not allow us to examine this low-velocity regime in more detail.

**Fig. 4.**
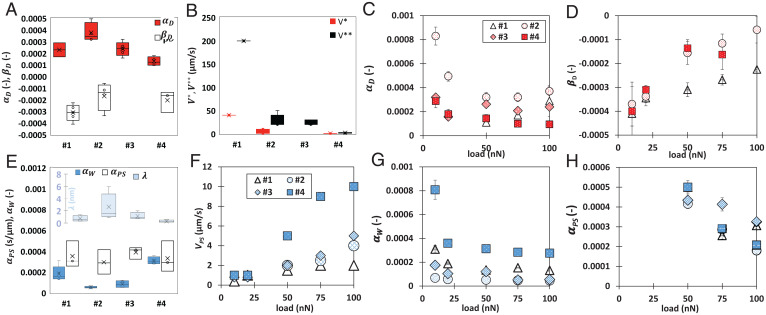
Fitting parameters for the friction of smooth and rough calcite single crystals in dry and aqueous environments. (*A*) Rate slopes $\alpha_{D}$ and $\beta_{D}$ in the velocity-weakening [D-I, $\beta_{D}\text{ln}(V)$] and velocity-strengthening [D-II, $\alpha_{D}\text{ln}(V)$] regimes, respectively, in dry nitrogen. Each box contains the results at all loads except for surface #2, which does not include 10 nN. (*B*) Transitions velocities $V^{*}$ and $V^{**}$ of each surface. Rate parameters (*C*) $\alpha_{D}$ and (*D*) $\beta_{D}$ as a function of load. (*E*) Rate parameters $\alpha_{PS}$ and $\alpha_{W}$ for regimes W-I and W-II, respectively, and correlation length $\lambda =kT/\alpha_{W}L$, all in an aqueous environment. (*F*) Transition velocity $V_{PS}$, (*G*) $\alpha_{W}$, and (*H*) $\alpha_{PS}$ as a function of normal load.

*SI Appendix*, Fig. S6 shows the single fits of the logarithmic functions to the experimental results to determine $\;V^{*}$ and $\beta_{D}$ as well as $\alpha_{D}$ and $V^{**}$. Fig. 4 and *SI Appendix*, Table S1 summarize the fitting parameters. The velocity-weakening frictional response of calcite is still prominent for rough calcite #2 (5-nm rms roughness) with $V^{*}$ ∼ 1 to 10 µm/s, while calcite #3 (10-nm rms roughness) does not exhibit a velocity-weakening regime in any replica experiment. Here, the plateau is very prominent at the highest loads as well as the velocity-strengthening friction at $V>V^{**}$. In contrast, the velocity-weakening friction of calcite #4 (15-nm rms roughness) is evident at velocities $<V^{*}$ ∼ 1 to 0.7 µm/s. Meanwhile, the velocity-strengthening response extends over a wider range of velocities with an increase in roughness: that is, $V^{**}$ is ∼200, 20 to 50, 20 to 30, and 1 to 3 µm/s for calcites #1, #2, #3, and #4, respectively. Hence, there is a shift of the transition between velocity-weakening and -strengthening friction to slower velocities (Fig. 4*B*).

The parameters $\alpha_{D}$ and $\beta_{D}$ are shown in Fig. 4*A*; each box includes the results at the investigated loads. Higher values of $\beta_{D}$ and $\alpha_{D}$ represent larger decrements and increments of friction with velocity, respectively, while values close to zero represent a trend to velocity-neutral behavior. There is a decreasing trend of $\alpha_{D}$ with an increase in rms roughness (average: 3.03× 10^−4^, 2.2 × 10^−4^, and 1.3× 10^−4^ for #2, #3, and #4, respectively). An increase in load (and stress) also leads to a decrease of $\alpha_{D}$ for these surfaces (Fig. 4*C*). The fitting range for the smooth surface is very narrow with very few data points, and hence, the estimated $\alpha_{D}$ for surface #1 is not further discussed. The slope in the velocity-weakening regime ($\beta_{D}$) is very close for surfaces #2 and #4 (Fig. 4*D*) and less negative than for the smooth surface (#1). An increase in load (stress) leads to a less negative slope of friction vs. velocity for all three surfaces #1, #2, and #4, indicative of a less significant velocity-weakening friction.

### Contact Aging and Memory Distance.

**Fig. 5.**
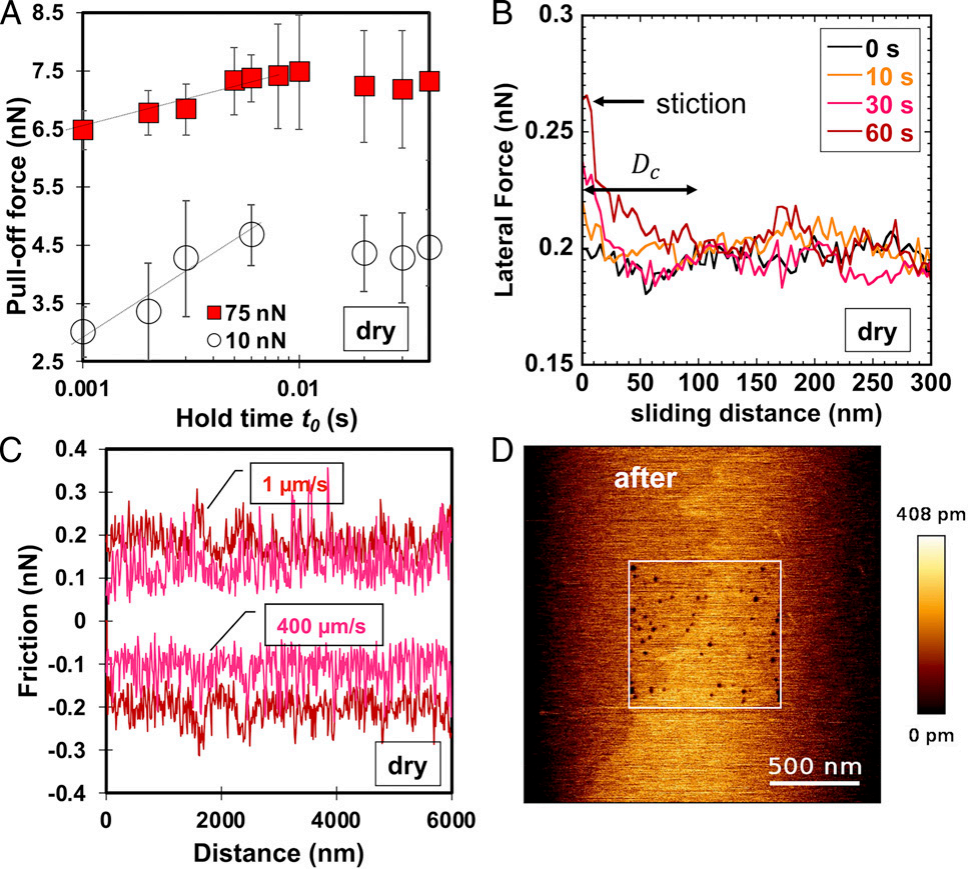
(*A*) Pull-off force vs. loading time for a smooth calcite surface in dry nitrogen. A sharp AFM tip was used to measure the pull-off force under the normal loads of 10 nN (black) and 75 nN (red). (*B*) Lateral force experienced by a blunt Si tip sliding at a velocity of 0.5 µm/s on smooth calcite after being held for a contact time between 0 and 60 s at 100 nN in dry nitrogen. (*C*) Representative stick–slip in friction loops on smooth calcite at sliding velocities of 1 µm/s (red) and 400 µm/s (pink) in dry nitrogen under a normal load of 100 nN. (*D*) AFM images (2 × 2 µm) of a smooth calcite surface after eight contact mode images (normal load of 75 nN and sliding velocity of 1 µm/s) were taken in the area limited by the white square (800 × 800 nm). The 2- × 2-µm images were taken in contact mode as well but at a small load of 5 nN to avoid wear of the surface.

Contact aging is a well-recognized phenomenon that is reflected in velocity-weakening kinetic friction as well as in a logarithmic increase of static friction and of the pull-off force with the static loading time (8). To examine the origin of the velocity-weakening friction in Fig. 3, pull-off force measurements were performed as a function of the static loading time on smooth calcite only, and the results are shown in Fig. 5*A*. The pull-off force increased weakly with the logarithm of the duration of the static loading at times longer than 1 ms and saturated beyond 10 ms. Each pull-off force value was determined from the average of 16 measurements at different locations of the calcite surface, which led to a large SD. Note that these measurements were repeated with other tips and calcite samples, and the results were qualitatively similar. Hence, the small logarithmic increase is considered to be significant. Additionally, static friction measurements were conducted on the smooth calcite surface by holding the load from ∼0 to 60 s before pulling the cantilever laterally. The friction loops showed a small stiction peak friction at times of >0 s (Fig. 5*B*), but the trend as a function of the loading time was not evaluated due to the concurrent change of topography. Imaging of the calcite surface where the friction measurements were conducted (the white square in Fig. 5*D* highlights the region where friction measurements were performed) showed the appearance of nanoscale pits during contact mode imaging (Fig. 5*D*) (after eight scans at an applied load of 75 nN). Prominent stick–slip was observed upon sliding in the dry environment (Fig. 5*C*). Contact mode images were also taken on roughened calcite #2. While it was more difficult to identify changes of topography compared with smooth calcite, the valleys between protruding grains became deeper during sliding; one example is shown in *SI Appendix*, Fig. S7.

Contact aging has been shown to originate from the atomic attrition of ionic crystals, like NaCl and KBr (34, 35). Atomic attrition refers to the removal and transfer of individual atoms across the contact interface. Gnecco et al. (35) observed the evolution of friction over thousands of scans along with the formation of scratches, pits, and mounds on the KBr surface and velocity-weakening friction in AFM measurements. In friction loops, the ion removal was recognized through the irregularity of the friction loops, where the number of displaced ion pairs was comparable with the number of slip events, as the ions were detached when the tip jumps from one stick position to the next one. Our results in Fig. 5 and *SI Appendix*, Fig. S7 support that the atomic attrition of the calcite surface can be the origin of contact aging and the velocity-weakening friction in Fig. 3. The increase of the load (stress) leads to a smaller negative slope $\beta_{D}$, likely because atomic attrition is promoted with an increase in stress, and hence, the effect of increasing velocity (i.e., the relevance of contact aging) is mitigated (Fig. 4*D*). The fact that $\beta_{D}$ is more negative for the smooth surface compared with #2 or #4 (rough) surfaces indicates that atomic attrition becomes much less significant on the smooth surface with an increase in velocity, likely due to the smaller applied pressures.

The memory distance $D_{c}$ connects contact aging and velocity-weakening friction in RSF constitutive equations. The memory distance has been assumed to be the distance required for a population of asperity contacts to renew itself, thus counteracting the effects of aging, and is thought to be equal to the average size of the population of asperities in contact. Based on that, contacts on the frictional interface have an effective contact time $D_{c}/V$. On smooth calcite, the transition velocity is $V^{*}∼$ 40 µm/s. Following the same methodology as in ref. 10 for silica–silica contacts, we estimate the memory distance with $V^{*}$ and the time required for the adhesion strength to increase after coming into contact (≤0.001 s). This yields 40 nm as the upper limit of $D_{c}$. The memory distance is in the order of the contact size between the tip and the smooth surface (∼15 nm at 100 nN) (*SI Appendix*, Table S2). Note that the minimum time required for contact aging could be smaller than 0.001 s, which would reduce the estimated value of the memory distance. Interestingly, the lateral force in Fig. 5*A* achieves a quasiplateau after sliding for ∼50 nm, thus close to the estimated maximum of $D_{c}$, and some works at the macroscale have related this distance to $D_{c}$ (36). Assuming that the mechanism underlying contact aging of the rough contacts is also atomic attrition with the same time constant (0.001 s), the smaller transition velocity $V^{*}$ with an increase in roughness would yield a smaller memory distance (i.e., 40, ∼1 to 10, and ∼0.7 to 1 nm for calcites #1, #2, and #4, respectively).

The fact that velocity-weakening friction is most prominent for surface #1 followed by #2 and vanishing for #3 is important. If the memory distance is related to the contact area, it is reasonable that it is smallest for #3 since 1) this surface exhibits the largest peak-to-peak distance (i.e., fewer asperities in the contact) and 2) the mean asperity radius is smaller than that of surface #4 (Table 1). We note that the velocity-weakening friction reappears on calcite #4 ($V^{*}$ ∼ 0.7 to 1 µm/s), which coincides with the smaller asperity–asperity distance of surface #4 compared with #3 and thus, a higher asperity number density within the contact. Presumably, the contact area of the tip with surface #2 upon sliding is larger than with surface #4, despite the smaller pull-off force (Fig. 2*A*), suggesting that noncontact interactions are responsible for the larger pull-off force on surface #4.

Importantly, based on the mechanokinetic model, the increase in friction with velocity at high sliding velocities arises when the aging phenomenon freezes, and damping effects start to dominate; the plateau results from the superposition of both phenomena with similar contributions to friction. Damping effects include nonaging contributions to friction, like phonon dissipation (10). In this model, the phonon dissipation would lead to a lineal increase of friction with velocity (37); we note that a linear fit would be equally possible for the smooth surface in the velocity-strengthening regime. On the other hand, an inherent interaction energy corrugation leading to local energy barriers to sliding (reflected in stick–slip or jumps of the cantilever) would introduce a logarithmic dependence on velocity. The logarithmic dependence is evident for the rough surfaces in the range of investigated velocities. However, the fact that $\alpha_{D}$ decreases with increasing roughness (*cf* calcites #2, #3, and #4 in Fig. 4*A*) and tends to zero suggests a decreasing trapping probability between tip and rough surfaces as a result of the modified surface topography.

### Friction in Aqueous Environments.

**Fig. 6.**
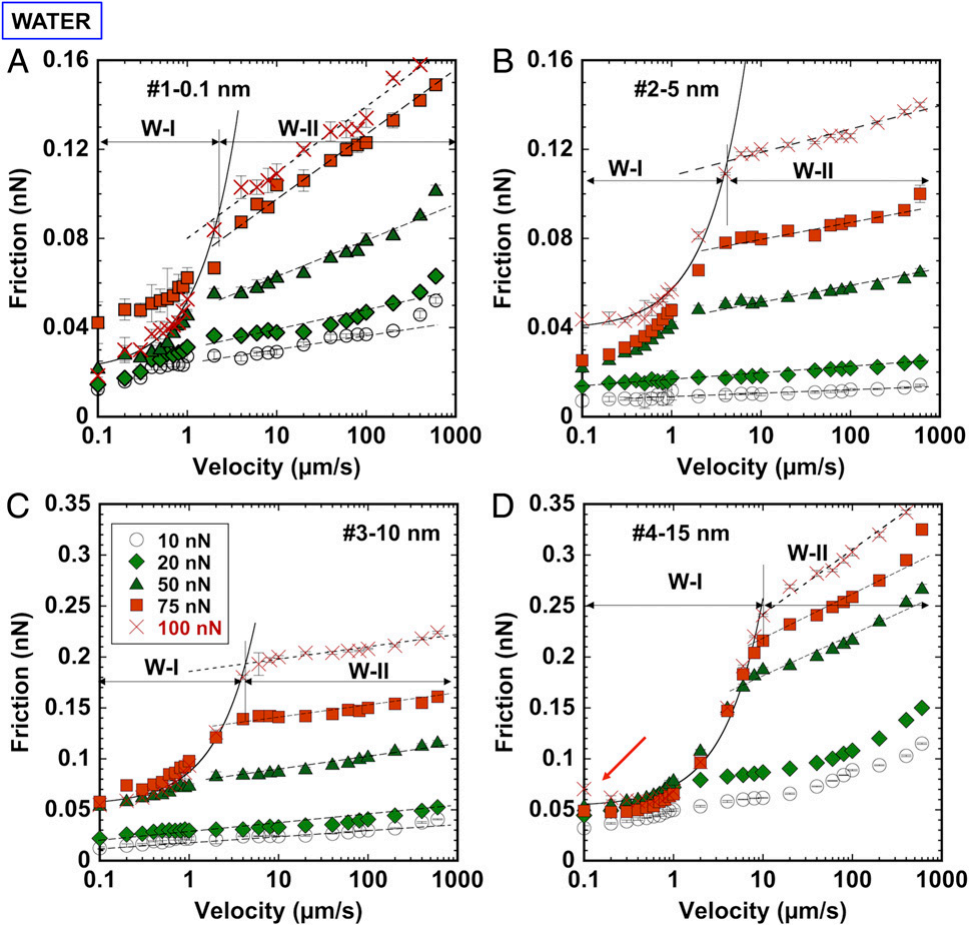
Friction measurements between (*A*) smooth or (*B*–*D*) rough calcite surfaces and a blunt Si tip as a function of velocity from 0.1 to 600 μm/s. Three different regimes are distinguished. At small loads (10 nN), friction increases with the logarithm of velocity. At higher loads, regime W-I extends to *V* < $V_{PS}$, where F∼V and the pressure solution is most prominent, and regime W-II (*V* > $V_{PS}$), where F∼lnV. Black dashed lines represent logarithmic fits to the friction force in regime W-II, and full lines represent the linear fit in regime W-I. Note the different scales of the *y* axes of the plots. The fits of the models to the experimental results are in *SI Appendix*, Fig. S11, and the fitting parameters are in *SI Appendix*, Table S3.

Friction measurements were conducted on smooth and rough calcite surfaces as a function of sliding velocity in an aqueous environment (Fig. 6). The reproducibility of the experimental results using different AFM tips is very satisfactory (*cf* Fig. 6 and *SI Appendix*, Fig. S8). Under all conditions, the lubricated contact is greatly nonadhesive (pull-off force <0.5 nN). In contrast to the dry contact, velocity-weakening friction is not observed, and stick–slip vanishes (*SI Appendix*, Fig. S9). The logarithmic trend $F_{L}∼\ln(V)$ is maintained only at high velocities (regime W-II, *V* > $V_{PS}$), while a clear deviation from this scaling relation is observed with a decrease in velocity (regime W-I, *V* < $V_{PS}$), and friction is much smaller than predicted by the logarithmic trend. This behavior was reported for smooth (freshly cleaved) calcite surfaces in equilibrium with a calcium carbonate–saturated solution as well as upon addition of CaCl_2_ and NaCl, and we attributed it to the onset of the pressure solution of calcite below a critical velocity based on thermodynamic and kinetic estimations (11). Calcite thus dissolves due to a local undersaturation of the solution resulting from the applied pressure on the crystal, and the dissolved calcium and carbonate ions hydrate and lubricate the contact between the two calcite crystals or between the calcite crystal and an AFM tip, which reduce the friction force, a mechanism that was called pressure solution–facilitated slip (13). The “cross-over” of the friction vs. velocity curves at the loads of 75 and 100 nN in regime W-I (Fig. 6*A*) indicates the decrease in friction with an increase of the applied load, thereby leading to an apparent negative coefficient of friction; *SI Appendix*, Fig. S10*A* shows the decrease of friction with load at selected sliding velocities. This is also a footprint of the pressure solution–facilitated slip since higher pressures accelerate the dissolution of calcite. Another important characteristic is that friction increases linearly with velocity (F∼V) in regime W-I (solid line in Fig. 6*A*). Although in our previous studies on smooth calcite crystals (11, 12), we did not notice this linear relation, we have confirmed that it describes those reported results as well.

Fig. 6 *B*–*D* shows qualitatively similar results for rough calcite surfaces with two distinct regimes, W-I and W-II. *SI Appendix*, Fig. S10 *B*–*D* shows friction as a function of the load for the lubricated rough contacts. In contrast to the dry environment, deviations from the linear relation are observed at $V<V_{PS}$ and at the highest loads (shadowed regions in *SI Appendix*, Fig. S10 *B*–*D*). This is a footprint for pressure solution–facilitated slip in W-I. Importantly, on the roughest calcite surface (#4) (Fig. 5*D*), a slight decrease in friction with velocity is observed in regime W-I (the red arrow in Fig. 6*D*), indicating that contact aging happens concurrently with pressure solution in this case. Interestingly, a deviation of friction from the logarithmic trend is often observed at the highest velocity at loads of 10 and 20 nN (*SI Appendix*, Fig. S11). This might reflect the liftoff of the tip and the transition into a different lubrication regime. Because the surface separation is unknown in AFM experiments, we do not further discuss this deviation.

#### Pressure solution–facilitated slip ($F_{L}∼V$ *at* $V<V_{\mathrm{PS}}$).

A linear relation between friction and velocity reflects the viscous dissipation of a Newtonian fluid based on Reynold’s law of lubrication (38). Although the hydrodynamic lift is negligible here, a disjoining pressure (Π)—arising from Derjaguin-Landau-Verwey-Overbeek (DLVO) and hydration forces—prevents direct contact between the two surfaces under an applied normal stress ($\sigma_{n}$) (39). The effective normal stress $(\sigma_{n}-P_{f}$) is thus balanced by the disjoining pressure in the confined fluid film, and the balance is achieved by adjusting the film thickness and/or fluid film composition (Fig. 2*C*). According to a recent molecular dynamics simulation study, the confined fluid film can sustain normal stresses higher than 1 GPa (40). In the context of hydration lubrication of two atomically smooth mica surfaces, the linear relation between friction and velocity was associated with the viscous dissipation in the sheared hydration shells of the ions in the confined film, with the linear relationship indicating that the film behaves as a Newtonian fluid (41). This is consistent with the phenomenon of pressure solution, as the dissolved ions and their hydration shell replenish the confined fluid film and make it more lubricious. Based on this, $\mu_{W-I}∼\mu_{PS,0}+\Omega \cdot \eta \cdot V$, with η being the viscosity of the trapped fluid between the two surfaces and Ω being a geometric factor. We choose $\alpha_{PS}=\Omega \cdot \eta$ to fit our results, which accounts for both the viscosity of the confined solution and the contact geometry. Fig. 4*E* includes the values of $\alpha_{PS}$ for 50, 75, and 100 nN. The similar values for the four surfaces support that the main mechanism underlying friction is the viscous dissipation upon shear of the fluid, and hence, it mainly depends on the properties of the confined liquid. These results also support that this fluid film is also maintained at the multiasperity contacts between tip and rough surfaces. Note that $\alpha_{PS}$ has units of seconds per micrometer, and hence, it is not directly comparable with $\alpha_{D}$ and $\beta_{D}$.

Fig. 4*F* shows the velocity $V_{PS}$ at the transition between regimes W-I and W-II for the four calcite surfaces. $V_{PS}$ increases from ∼1 to ∼2, 4, 5, and 10 µm/s with increases in load from 10 to 100 nN on #1, #2, #3, and #4 calcite surfaces, respectively, which indicates that less contact time is required for the pressure solution to happen with an increase in rms roughness. In other words, pressure solution–facilitated slip is promoted by roughness. This should be due to the convoluted influence of the stress distribution in the multiasperity contacts, the effect of the surface topography on the fluid confinement, and the composition of the trapped fluid. It is also possible that the nanoscale roughness of the asperities (not resolved in the AFM images) further promotes dissolution.

#### Thin-film lubrication and shear thinning ($F_{L}∼\ln(V)$ *at* $V>V_{\mathrm{PS}}$).

The logarithmic relation between friction and velocity can be modeled as the shear-promoted thermally activated slip of the surface-localized hydrated ions in the context of transition-state theory (42). This theory was derived by Eyring (43) to describe liquid viscosity and shear thinning at the molecular level based on activated flow. According to this model, for slip to occur, the liquid molecule (hydrated ions here), initially in an equilibrium position (an energy minimum), needs to jump over a “transition state” before reaching the adjacent energetic minimum, which requires an activation energy Q′. Although the thermal energy of the molecules might be sufficient to overcome this energy barrier, the shear stress applied on the molecule reduces the energy barrier and thereby, promotes slip. When the molecule falls in the adjacent minimum, the applied work is irreversibly dissipated, which is the origin of friction. Following the approach of He et al. (44), the friction force is given by

[3] $$F_{L}=F_{0}+\frac{k_{B}T}{\lambda }\text{ln}(V),$$

where $F_{0}=\frac{A}{\phi }\left(Q^{`}+P\theta -k_{B}T\;\text{ln}(V_{0})\right)$, λ=ϕ/A is a coherence length, $V_{0}$ is the reference velocity above which the thermal activation vanishes, $k_{B}$ is the Boltzmann constant, T is the absolute temperature, P is the normal pressure, ϕ is the shear activation volume, and θ is the pressure activation volume. The parameter λ was obtained by fitting Eq. **3** to the experimental results and is shown in Fig. 4*E*. Previous works (44) have interpreted λ as the molecular coordination in a confined thin film; higher coordination has been related to a more prominent fluid structure and to the collective motion of several molecules, which lowers friction. Note that λ is not strictly the same as the shear activation length defined at the molecular level in Eyring’s model to describe viscosity and shear thinning.

We also define a friction coefficient in regime W-II (*V* > *V_PS_*) (*SI Appendix*, Fig. S12):

[4] $$\mu_{W-II}=\mu_{W,0}+\alpha_{W}\ln(V),$$

whereby $\alpha_{W}=k_{B}T/(\lambda \cdot L)$ and $\mu_{W,0}=F_{L}/L$.

Interestingly, λ increases when the rms roughness increases from 0.1 to 5 nm (#1 vs. #2), and it gradually decreases with an increase to 10- and 15-nm rms roughness (#3 vs. #4). An inverse (nonmonotonic) trend is observed for $\alpha_{W}$ (Fig. 4*E*), with the highest values obtained for calcite #4. Considering the opposite change of $\alpha_{W}$ compared with $\alpha_{D}$, the change of $\alpha_{W}$ with roughness should be mainly due to the influence of the fluid film. The increase (decrease) of λ ($\alpha_{W}$) from #1 to #2 might result from the reduced interaction between the two surfaces due to roughness (45), leading to thicker films and larger correlations lengths. It is possible that larger roughness reduces the interfacial ordering and disturbs the molecular coordination, thereby justifying the decrease in λ. The decrease of λ with an increase in load (stress) can be related to the thinning of the fluid film as fluid is squeezed out, which reduces the molecular coordination (*SI Appendix*, Fig. S12). Note that the fits of the model to the experimental results at 10 nN for calcites #3 and #4 are not included in this plot since the logarithmic fit does not lead to acceptable results (*SI Appendix*, Fig. S11), indicating a transition of the behavior.

Fig. 4 *G* and *H* shows the decrease of $\alpha_{W}$ and $\alpha_{PS}$ with normal load, indicating the evolution toward a velocity-neutral friction coefficient with an increase in stress (*SI Appendix*, Table S2) both in the presence and in the absence of pressure solution. Decreasing values of a−b with normal stress above 50 MPa have been also reported for gouge of Carrara marble under water-saturated conditions (46) and attributed to a transition from brittle to ductile behavior mainly due to dissolution and precipitation and to plastic deformation at low velocities and high stresses. While our results agree in that calcite dissolution is related to the decrease of $\alpha_{PS}$ with stress, our analysis also shows that the properties of the confined fluid film at high confining stresses can be related to the decrease of $\alpha_{W}$ with stress.

### Comparison of Friction in Dry and Aqueous Environments at Single- and Multiasperity Contacts.

**Fig. 7.**
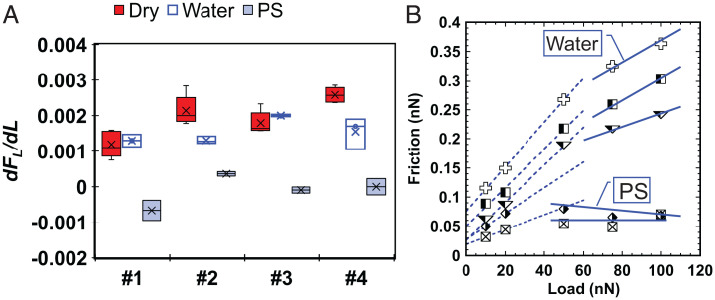
(*A*) Friction coefficient in the dry environment and in the two regimes of the aqueous environment at low loads (labeled as water) and high loads (labeled as PS). Each box includes results at velocities of 0.1, 1, 10, 100, and 600 µm/s. (*B*) Friction vs. load for calcite surface #4 at various velocities (0.1, 1, 10, 100, and 600 µm/s) illustrating how the slope of friction vs. load ($dF_{L}/dL$) in the aqueous environment was obtained in two regimes at low and high loads (labeled as water and PS in *A*, respectively). The friction vs. load curves for other conditions are shown in *SI Appendix*, Figs. S5 and S10.

Fig. 7*A* shows the estimated friction coefficients in dry and aqueous environments. The relation between friction and load for rough calcite–tip contacts is linear in dry environments, and it is roughly fit by a linear regression for smooth surfaces (*SI Appendix*, Fig. S5). In contrast, a clear deviation from a linear relationship is observed in aqueous environments (Fig. 7*B* and *SI Appendix*, Fig. S10). A similar decrease in slope with load and even negative slopes were observed in our previous measurements on smooth calcite in aqueous solutions (11, 39) and attributed to pressure solution–facilitated slip; yet, this is an observation on multiasperity contacts. To quantify the friction coefficient in the lubricated contacts, we have considered two different linear regimes and determined the corresponding slopes $dF_{L}/dL$ at low and high velocities, labeled as “aqueous” and “PS” friction coefficients, respectively. Fig. 7*A* illustrates that aqueous lubrication leads generally to a significant decrease in friction $\mu_{W}<\mu_{D}$, especially if pressure solution happens ($\mu_{PS}<\mu_{W}$), yet the reduction is more dramatic at rough than smooth contacts. This suggests that these trends could still apply at larger (multiasperity) contacts. The dry friction coefficient is smallest for the smooth surface (#1), as expected from the interlocking asperity model (47). That is, energy is dissipated when the tip hits the asperities and overcomes the compressive external load and the attractive intermolecular interactions as it raises the asperities. We observe that $\mu_{D}$ for #3 is smaller than for #2 and #4; this might be due to the larger asperity–asperity distance, which reduces the number of collisions of the tip with asperities. In this case, $\mu_{W}\sim \mu_{D}$, which suggests that the aqueous film might not be fully sustained between the asperities, perhaps due to the small contact area and thus, higher pressure compared with other surfaces. Nevertheless, pressure solution still happens, as inferred from the small $\mu_{PS}$ on surface #3.

We attempt now to cautiously extrapolate our results at room temperature to carbonate rock/gouge friction and seismicity at shallow crustal levels. A recent study (48) agrees with our results in that pore fluids lower the fault strength (friction coefficient) compared with dry conditions. This leads to an accelerated weakening as a result of faster subcritical crack growth and intergranular lubrication. Our findings are also consistent with very low–frequency earthquakes in the presence of pore fluids (49). These laboratory results suggest slip weakening (i.e., the decrease of the friction coefficient while sliding at constant velocity) to be a potential mechanism for such slow earthquakes. Note that the pressure-induced dissolution of more and more asperities could explain this gradual decrease of the friction coefficient at the macroscale.

We note the significantly smaller friction coefficients in Fig. 7 compared with macroscale friction of fault gouge and rocks (0.6 to 0.7). This is not a unique observation for calcite, but it has been often reported that the friction coefficient is scale dependent, and it decreases from the macroscale to the nanoscale for crystalline surfaces (50). In the larger contacts of fault gouge and rocks, the larger number of asperities undergoing plastic deformation and wear, the more frequent asperity interlocking, the larger number of particles trapped at the interface enhancing the third-body plowing contribution to the friction force, and granular flow can cause a significant increase in friction compared with nanoscale experiments.

There are abundant reports on the friction rate parameter  a–b of the RSF equation (Eq. **2**) for carbonate fault materials. Reported a–b values for dry friction range between −0.0036 and 0.03 for a variety of carbonate rock and gouge (33, 51, 52). Note that we can only compare the RSF parameters with $\beta_{D}$ because the velocity-strengthening friction observed in AFM experiments is not seen at the macroscale. At the nanoscale, $\beta_{D}$ falls in the range from ∼0 to ∼−0.0004, and hence, it is at least approximately two orders of magnitude smaller. The weaker contact aging in our experiments might rely on the restricted plastic deformation. Under water-saturated conditions, friction measurements with powdered gouge of Carrara marble showed a transition from velocity-strengthening to velocity-weakening friction with an increase in velocity and values of a–b between −0.005 and 0.016, while at stresses smaller than 20 MPa, the behavior was velocity independent (46). For pure calcite gouge, the reported a–b parameter ranges from −0.005 to 0.0025, with more prominent velocity-weakening behavior at 5 MPa and room temperature (53). In aqueous environment, we mainly observe velocity-strengthening friction, and the friction rate parameter $\alpha_{W}$ is about one order of magnitude smaller. Given the increasing trend of $\alpha_{W}$ with roughness and the decreasing trend with load (stress), it is possible that the higher roughness of gouge and rocks and the smaller normal stress justify the difference between the model parameters.

In aqueous environments, velocity-weakening friction was only observed for the calcite surface with the highest roughness at 100 nN. While more studies are needed, we hypothesize that this might be related to the induced precipitation of calcite at the contact during pressure solution. In fact, our SFA studies on calcite single crystals showed that the kinetic friction increased while sliding took place with concurrent precipitation, which indicated certain strengthening (cementation) of the contact during precipitation. Since the bulk solution is saturated with respect to calcite, the excess of ions in the confined fluid film may stem from the previous stress-induced dissolution of calcite, so that the dissolved mineral remains trapped in the confined fluid phase and leads to a local supersaturation. It is possible that smooth contacts are not able to trap the fluid and that roughness above a critical value is needed for this mechanism to play a role at multiasperity contacts. We note that reprecipitation of calcite after its pressure-induced dissolution has also been related to contact healing and overall gouge compaction (54) and to an increase in the friction coefficient (46) at the macroscale, and hence, our nanoscale studies provide a potential scenario for the velocity-weakening friction and friction instability at the nanoscale.

The decrease of the friction coefficient at nanoscale multiasperity contacts in aqueous environments and especially, when pressure solution happens is in qualitative agreement with the abundant macroscale evidence suggesting that fault frictional strength is weakened in the presence of reactive fluids (55–58) [e.g., due to mineral dissolution, chemical alteration, or transformation of fault minerals to secondary minerals at fault interfaces (57)]. Once slip commences, classical RSF equations would predict a decrease of the critical fault stiffness due to the fluid overpressure compared with dry conditions, thereby promoting aseismic slip. Assuming the validity of the RSF equation in the presence of water, we emphasize that not only the stress decreases but also, the friction rate parameters can vary based on our experimental results (*cf* $\alpha_{D}$ and $\alpha_{W}$ or $\beta_{D}$ and $\alpha_{PS}$); note that the relation between friction and velocity evolves from logarithmic to linear at the nanoscale, but how to extrapolate these results to the macroscale is still unknown. Based on our results, $D_{c}$ depends on the topography of the contact, which undergoes a complex evolution due to pressure solution (13). Hence, although a precise prediction of the critical stiffness is not possible yet, we recognize that both a reduction and an increase of critical stiffness are possible in the presence of fluids. This should be further investigated in the future.

In conclusion, we have examined the frictional behavior of single- and multiasperity contacts between tip and smooth and rough calcites, respectively, both in dry and aqueous environments. Two regimes of opposite logarithmic dependence of friction with velocity were observed in dry nitrogen. For the investigated system, contact aging due to atomic attrition was found to be responsible for the velocity-weakening friction and was most prominent on the smooth contacts. The size of the memory distance was estimated to be of the order of the contact size. Our work extends the mechanism of pressure solution–facilitated slip to rough contacts with calcite. The linear decrease of friction with decrease in velocity revealed the efficient lubrication of single- and multiasperity contacts when pressure solution of calcite happened. Given that fault materials with velocity-weakening properties are prone to promote earthquake nucleation based on the RSF constitutive equations, it is important that the fluid greatly eliminated the contact aging of dry smooth and rough contacts, but a scenario was identified where velocity-weakening friction remained. There are several aspects that need further investigation, like the velocity-weakening friction of rough contacts in an aqueous environment, high-temperature effects, and the contribution of the plastic deformation of calcite.

## Materials and Methods

### Sample Preparation.

To obtain calcite surfaces of different roughness, pieces of calcite cleaved along the ($10\bar{1}4$) plane were glued onto the sample holder of an Allied Multi-Prep system with DiamondBond glue. The other side of the calcite crystal was polished with diamond lapping films (8″ Disk; Allied High Tech Products, Inc.). The calcite surfaces labeled as #2, #3, and #4 were polished for 30 min at a rate of 50 rpm using 0.5-, 1-, and 3-µm diamond paper, respectively. The cleaved calcite surface (unpolished) is labeled as #1. For the experiments in equilibrium with an aqueous solution, calcium chloride (purity ≥ 99.0%; Sigma-Aldrich) was dissolved in nanopure water to achieve a concentration of 1 mM and then equilibrated with excessive calcite powder (purity ≥ 99.0%; Sigma-Aldrich) at room temperature. The unadjusted pH at equilibrium was constant and equal to 8.06.

### AFM Imaging.

Images of the roughened and freshly cleaved surfaces of calcite single crystals were taken in tapping mode at a scan rate of 1 Hz in dry nitrogen (∼0.4 psi) using a NanoWizard AFM (JPK) and a gold-coated tapping mode tip (Tap300GD-G; normal spring constant = 40 N/m, resonance frequency = 300 kHz; Budget Sensors). Tips were soaked in pure ethanol for 30 min and cleaned with ultraviolet ozone for at least 50 min prior to use. The rms roughness, mean curvature radii, and peak-to-peak distances were determined over areas of 1 × 1 µm and 500 × 500 nm using the Mountains9 software.

### Friction Measurements.

Friction measurements were carried out using silicon tips (CSC37/No Al; normal spring constant of 0.3 to 0.8 N/m; Mikromasch) that were thermally annealed for 2 h at 1,050 °C to increase their radius. While reproducible results were obtained with different tips (*SI Appendix*, Figs. S4 and S8), the radius of the tip used for the measurements shown in the manuscript is 190 nm as determined by scanning electron microscopy (SEM) imaging (*SI Appendix*, Fig. S1), and the spring constant is 0.38 N/m as determined by the thermal calibration method (59).

In friction measurements, the AFM tip slides a defined distance (6 µm) in a reciprocating fashion along the calcite surface at the selected constant load and velocity. When the AFM tip slides on the surface, it experiences a lateral force due to the friction force between tip and calcite, which leads to a lateral deflection of the cantilever. The lateral force is determined during both trace and retrace, with the lateral deflection and the lateral spring constant obtained by a noncontact thermal noise–based calibration method (60). The kinetic friction force is calculated as half of the difference between the lateral force during trace and retrace for each single friction loop. The friction force reported in this work was calculated as the average kinetic friction of 8 to 10 friction loops at each selected applied normal load and velocity with the error bar showing the SD. The normal load is defined as the force applied by the AFM cantilever on the substrate in the normal direction. The smallest friction force that can be accurately measured with our AFM is 0.01 nN. The kinetic friction measurements were performed at applied loads of 10, 20, 50, 75, and 100 nN, and the sliding velocity was varied in the range from 0.1 to 600 µm/s at each load. Once the area for the measurements was selected, a different spot within that area was selected for the measurement at each load. Static friction measurements were performed on a smooth calcite surface by varying the loading time between 0 and 60 s before pulling the cantilever laterally at a constant velocity of 0.5 µm/s and an applied load of 100 nN.

For friction measurements in a dry environment, a rubber ring was attached to the tip holder to seal the AFM cell, which was purged with a gentle dry nitrogen stream. The measurements in aqueous solution were performed in a homemade fluid cell, where the calcite single crystal was immersed in the solution and equilibrated for 24 h before the measurement. Note that the saturation of the solution with calcium carbonate prior to this equilibration step minimizes dissolution of the calcite crystal. The fluid cell was covered by a membrane to prevent evaporation during measurements.

To evaluate the potential wear of the tips, we measured the pull-off force between the tip and an Si wafer in a dry nitrogen atmosphere before and after friction measurements to estimate the change of the tip radius using the DMT contact mechanics model (in the next section) as any change of tip radius would be reflected in a change of adhesion. The assumption of a Hamaker constant of 6.7 10^−20^ J for a silica–silica contact (61), considering the native oxide layer of the Si tip and an Si wafer, yields a work of adhesion of 0.101 N/m, which was used in the DMT model. In addition to this, the tips were imaged at the end of the AFM experiments by SEM to measure the tip radius more precisely. One of the reasons why wear is reduced is that the tips were thermally annealed, which significantly increased the tip radius, decreased the applied pressure, and smoothened the tip surface.

### Pull-Off Force Measurements.

To rule out the plastic deformation of the asperities in the range of applied loads, pull-off force measurements were carried out on smooth and rough calcite surfaces with a blunt tip in a dry nitrogen environment as a function of the normal load (10 to 100 nN). Ten replica measurements were taken on each load to determine the average pull-off force on each calcite surface.

The pull-off force was also measured upon retraction of the tip after maintaining the load constant for a period ranging between 0 and 10 s to evaluate the potential role of contact aging. Two normal loads were selected, 10 and 75 nN, on smooth calcite surfaces in a dry nitrogen environment.

### DMT Model of Contact Mechanics.

Because the pull-off force between the tip and smooth calcite surfaces in dry nitrogen was shown to be nonnegligible, the DMT model was applied to determine the contact stress. The DMT model assumes a smooth contact and that the contact profile remains the same as in a Hertzian contact with additional attractive interactions acting outside the area of contact. The work of adhesion was obtained from the pull-off force $\;F_{0}=-2\Delta \gamma \pi R$ to be Δγ = 44.3 ± 0.85 mN/m. The contact radius a is given by

[5] $$a={\left(\frac{3R}{4E^{*}}\left(L+2\Delta \gamma \pi R\right)\right)}^{\frac{1}{3}},$$

where $E^{*}$ is the reduced elastic modulus of the AFM tip and calcite and is given by

[6] $$\frac{1}{E^{*}}=\frac{1-v_{1}^{2}}{E_{1}}+\frac{1-v_{2}^{2}}{E_{2}},$$

with *E*_1_ = 84 GPa (62) and *E*_2_ = 130 GPa (27) being the Young’s modulus and *v*_1_ = 0.32 (63) and *v*_2_ = 0.28 (−) (27) being the Poisson’s ratio of calcite and silicon tip, respectively. To estimate the stress at the single asperity–tip contact, we assumed spherical and smooth asperities, with the arithmetic mean of the peak radius r obtained from the analysis of the surface roughness (Table 1), leading to an effective radius:

[7] $$1/R=1/R_{\mathrm{tip}}+1/r.$$

Note that the DMT model only applies strictly to smooth surfaces, and hence, deviations from the model are possible. The same model was also applied to contacts between Si tips and Si wafers to determine potential changes of the tip radius.

## Supplementary Material

Supplementary File

## Data Availability

All study data are included in the article and/or *SI Appendix*.
