# Supplementary material for: Velocity-weakening and -strengthening friction at single and multiasperity contacts with calcite single crystals
Source: Proc Natl Acad Sci U S A. 2022 May 25;119(22):e2112505119. doi: 10.1073/pnas.2112505119 (PMC9295777; doi:10.1073/pnas.2112505119)
Supplement: Supplementary File [file pnas.2112505119.sapp.pdf]

PNAS
www.pnas.org

Supplementary Information for

Velocity-weakening and -strengthening friction at single and multi-asperity contacts with calcite single crystals

Binxin Fu[1], and Rosa M. Espinosa-Marzal[1,2*]

1 Department of Civil and Environmental Engineering, University of Illinois at Urbana-Champaign, 205 N. Matthews Avenue, Urbana, IL 61801, United States.

2 Department of Materials Science and Engineering, University of Illinois at Urbana-Champaign, 1304 W. Green St., Urbana, IL 61801, United States

*Rosa M. Espinosa-Marzal, Tel: +1 217 607 3856, Email: rosae@illinois.edu

This PDF file includes:

Figures S1 to S12
Tables S1 to S3

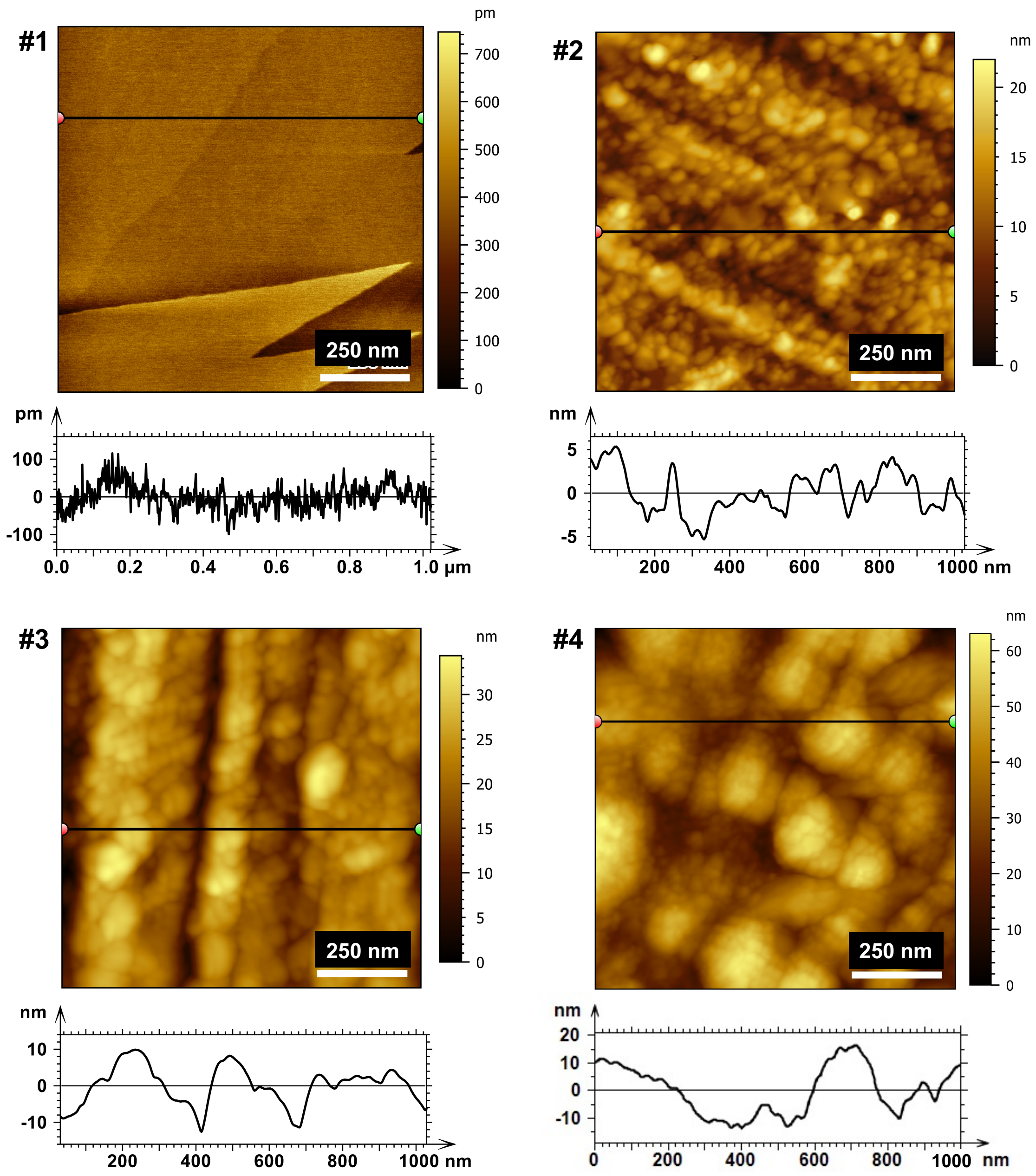

Figure S1. Representative 1 µm × 1 µm images and cross section topography of calcite surfaces #1, #2, #3 and #4 taken by AFM tapping mode.

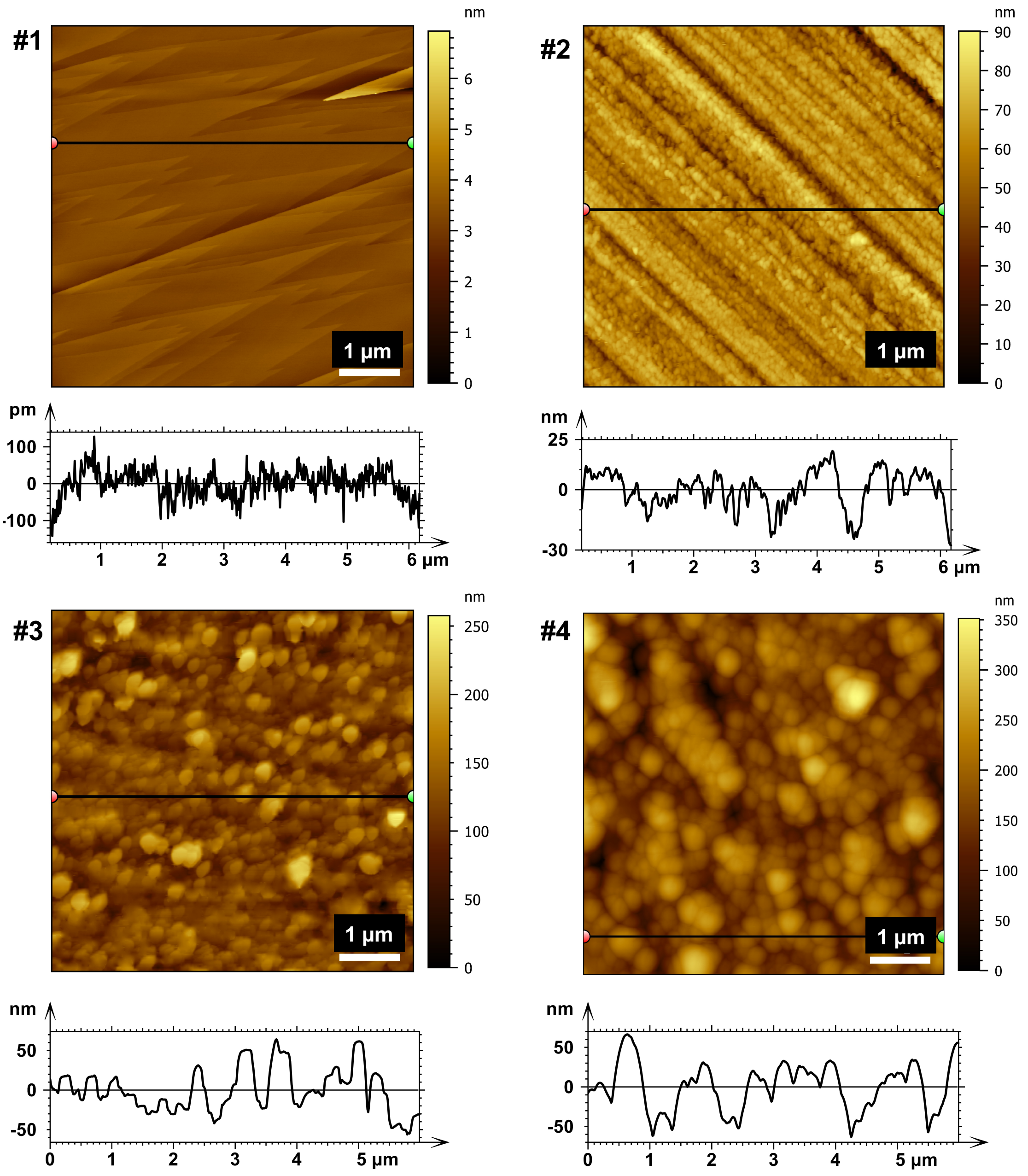

Figure S2. Representative (a-d) 6 µm x 6 µm and cross section topography of calcite surfaces #1, #2, #3 and #4 taken by AFM tapping mode.

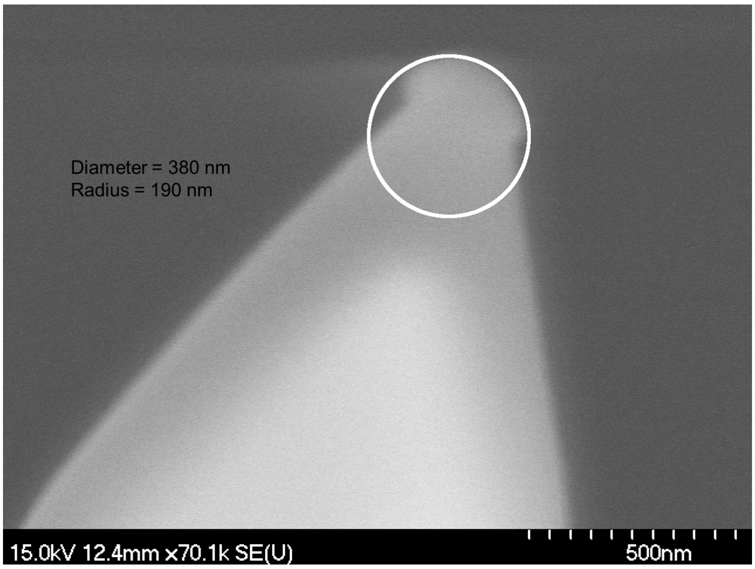

Figure S3. SEM image of the blunt AFM tip used in friction and pull-off force measurements shown in the manuscript. To examine the change of the tip geometry, the pull-off force was measured on a silicon wafer in dry nitrogen during the series of AFM measurements shown in the manuscript. Initially, the pull-off force was 38.5 $\pm$ 1.5 nN and after the conclusion of this set of friction measurements, the pull-off force was 46.6 $\pm$ 1.9 nN. A small increase of the pull-off force was indeed observed, which indicates that the tip radius increased by 15%. Such change in tip radius leads to a maximum decrease of contact stress of 8% on the smooth surface and changes smaller than 2% for the single asperities of the rough surfaces.

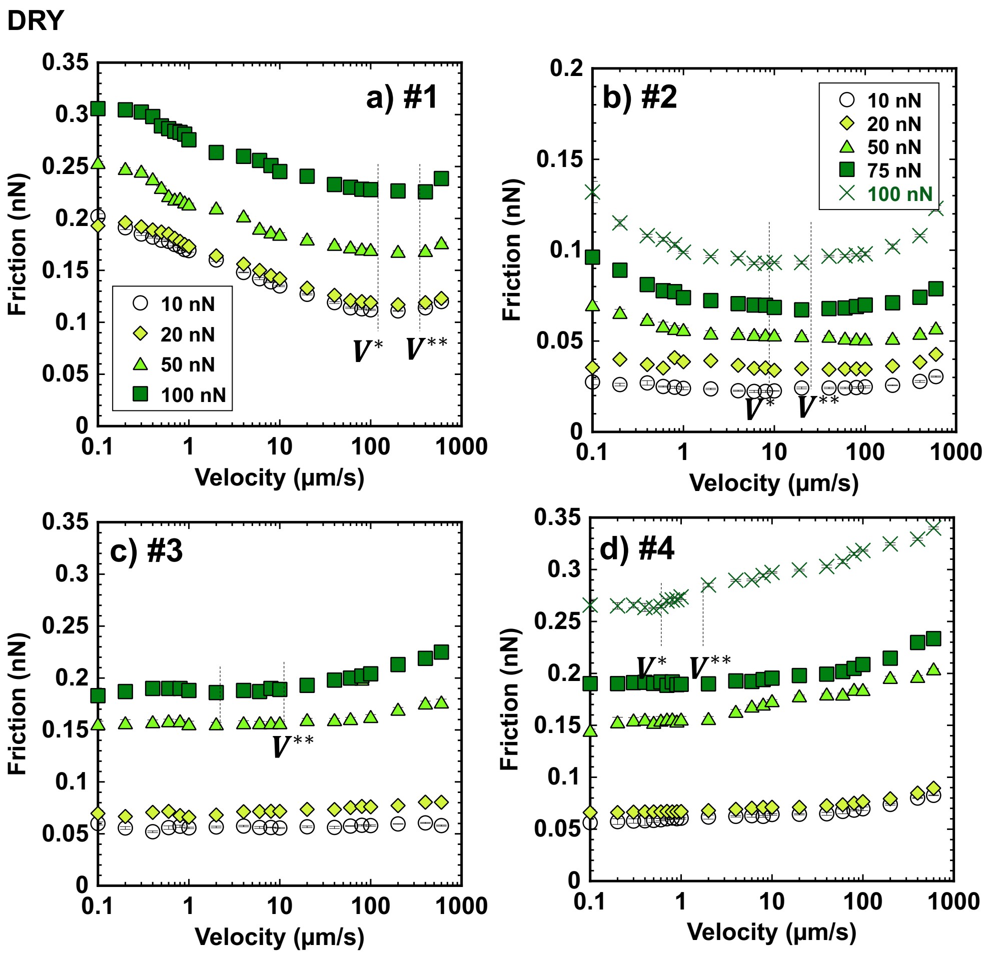

Figure S4. Friction force measurements on calcite surfaces #1, #2, #3 and #4, performed with two AFM tips (different from that used for the measurements shown in Figure 3). The error bars show the standard deviation, which is often smaller than the marker size and hence not visible. Tip 1 was used for calcite #1 and #4 (R~260 nm), and tip 2 was used for calcite #3 and #4 (R~141 nm).

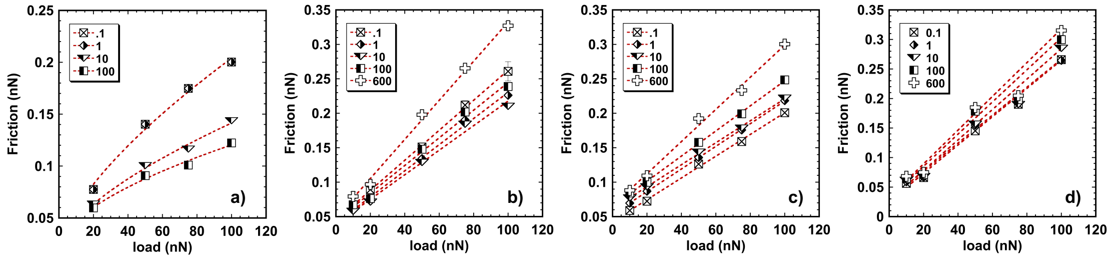

Figure S5. Friction as a function of load in dry nitrogen at investigated velocities of 0.1, 1, 10, 100 and 600 µm/s on a) #1, b) #2, c) #3 and d) #4 calcite surfaces. The relation between friction and load deviates from a linear relationship for #1. The exponent of the power law L^n used for the fits is 0.67, 0.67, 0.64 and 0.57 for 0.1, 1, 10 and 100 µm/s, respectively. This is attributed to the adhesive nature of the single asperity contact (1). The linear relationship for the rough surfaces supports the existence of multi-asperity contacts.

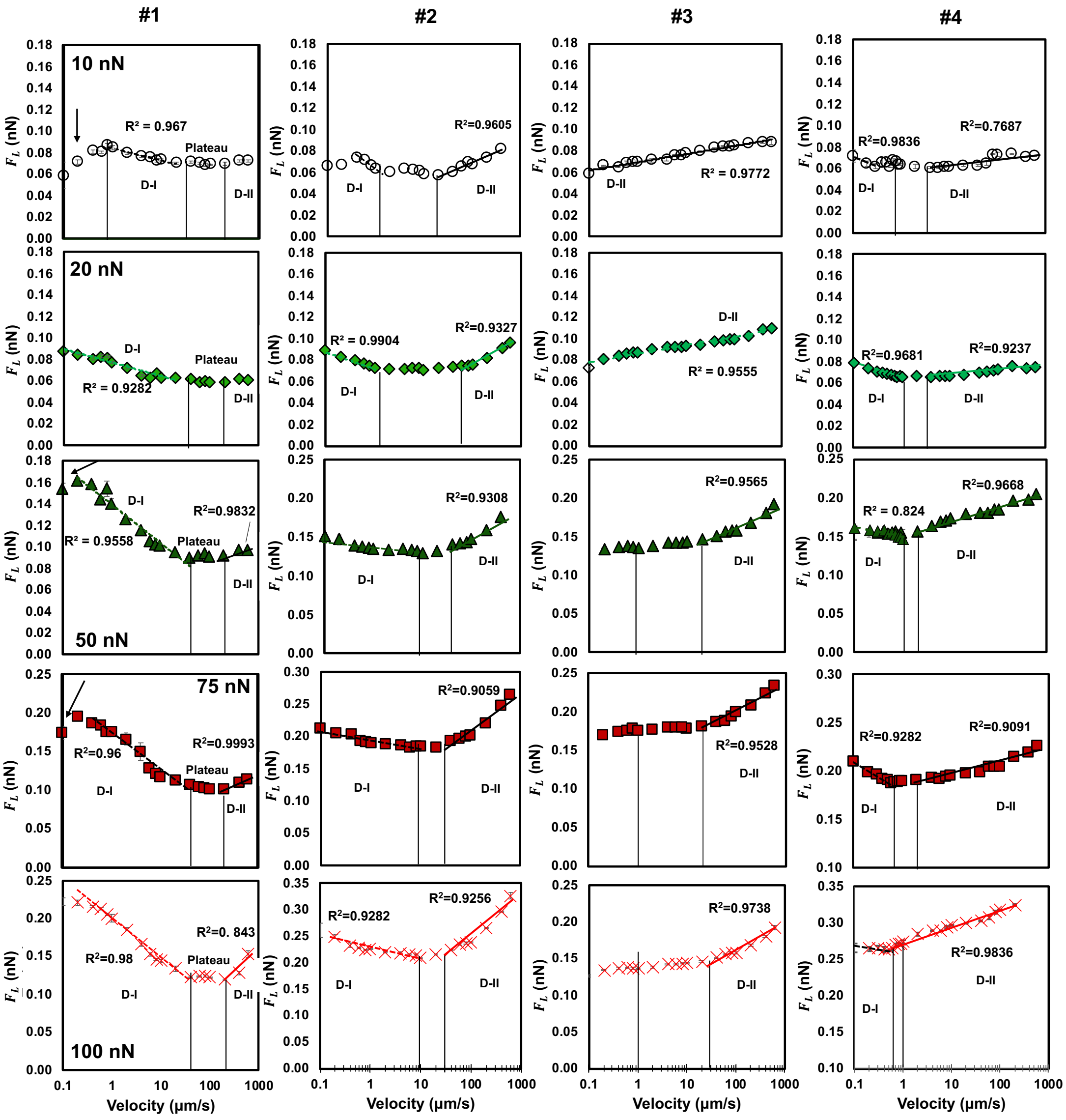

Figure S6. Friction force between the AFM tip and calcite. Each plot represents the results at a single normal load for calcite #1 (column 1), #2 (column 2), #3 (column 3) and #4 (column 4) and 10 nN (first row), 20 nN (second row), 50 nN (third row), 75 nN (fourth row) and 100 nN (fifth row). The transition velocities $V^{*}$and V^{**} and friction rate parameters α_D and β_D are summarized in detail in Table S1. The logarithmic fit for velocity weakening friction is shown by a dash line, while the fit to velocity strengthening friction is given by a solid line. Note that the y axis is not same for each plot.

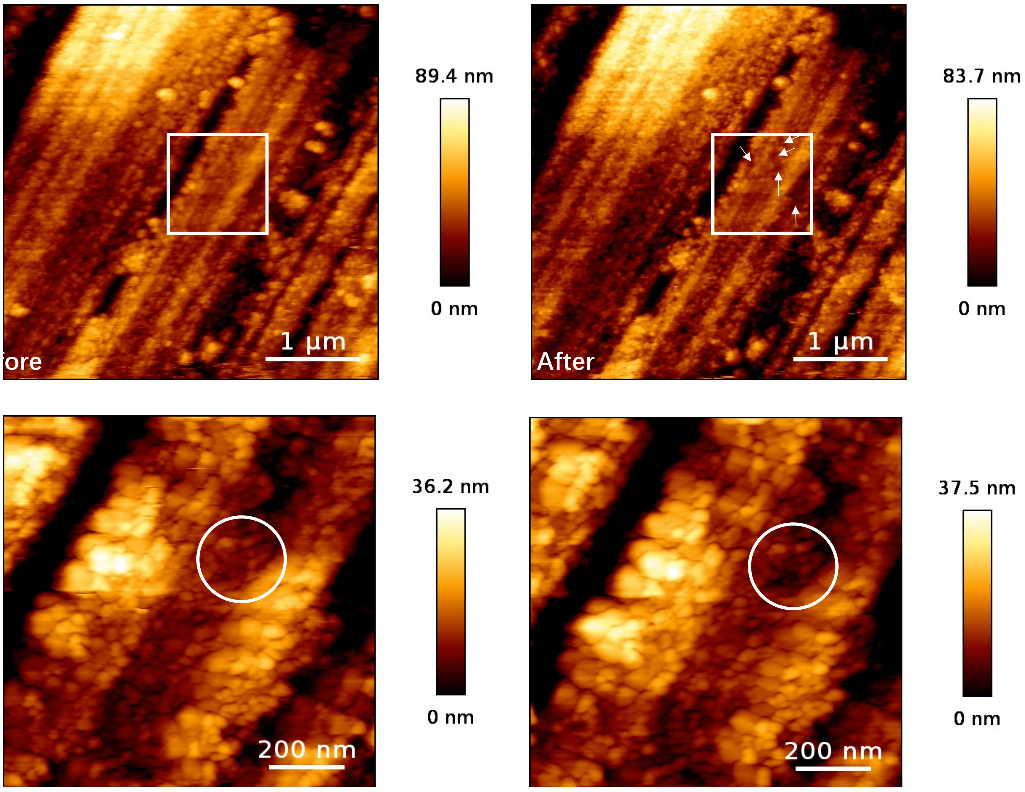

Figure S7. Contact mode images of calcite #2. (a-b) 5 µm x 5 µm images were taken at a set point of 5 nN, whereas the area within the white square (1 µm x 1 µm) was imaged at a set point of 50 nN for ten times and at 75 nN for eight times. The arrows point at some regions where wear was evident. Changes of topography were more difficult to visually identify due to the roughness. (c-d) Higher resolution images (1 µm × 1 µm) of the limited region in white square. The asperities in the white circle are removed during the contact mode imaging at high applied force.

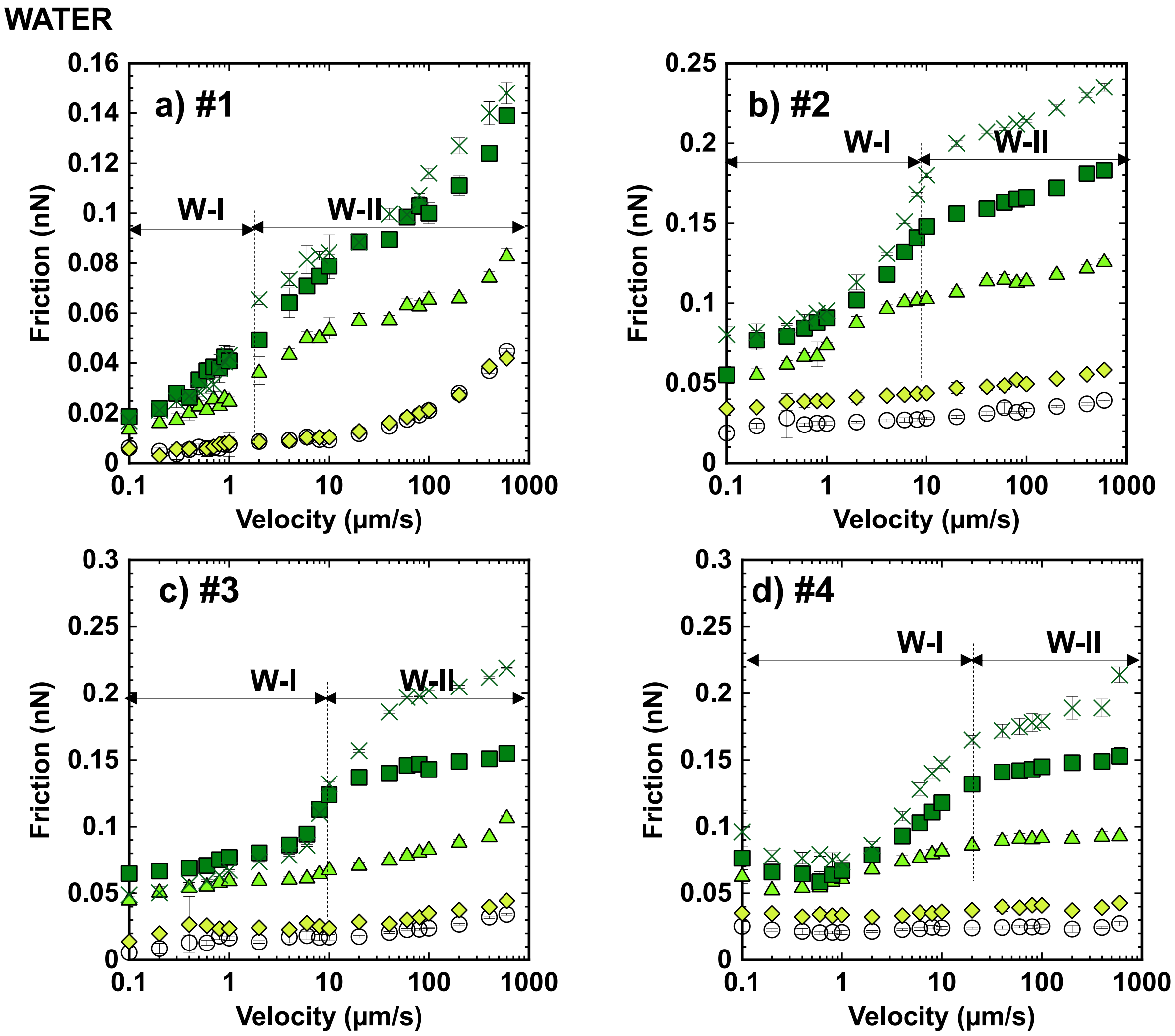

Figure S8. Additional friction force measurements on calcite surfaces #1, #2, #3 and #4, measured with three different tips in water. The error bars show the standard deviation, which is often smaller than the marker size and hence not visible. Tip 3 was used for calcite #1 (R~190 nm), tip 4 (R~276 nm) was used for calcite #2 and #4 and tip 5 (R~394 nm) was used for calcite #3.

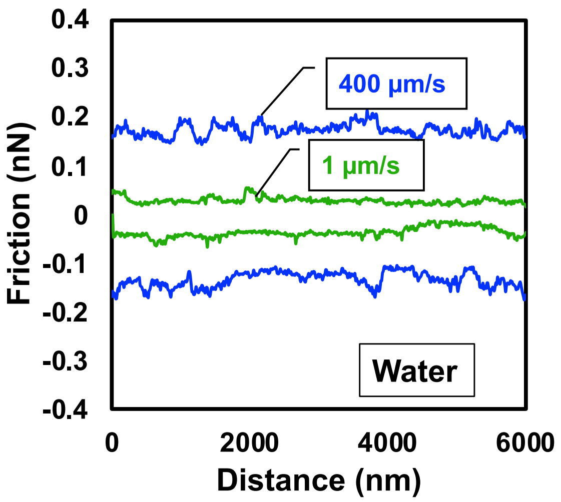

Figure S9. Representative friction loops on smooth calcite at a sliding velocity of 1 µm/s (green) and 400 µm/s (blue) in aqueous environment under a normal load of 100 nN. The friction measurements at 1 µm/s and 400 µm/s fall in the W-I and W-II regimes, respectively. The comparison with dry condition (Figure 5C in the main text) suggests that sliding in aqueous solution significantly eliminates stick-slip.

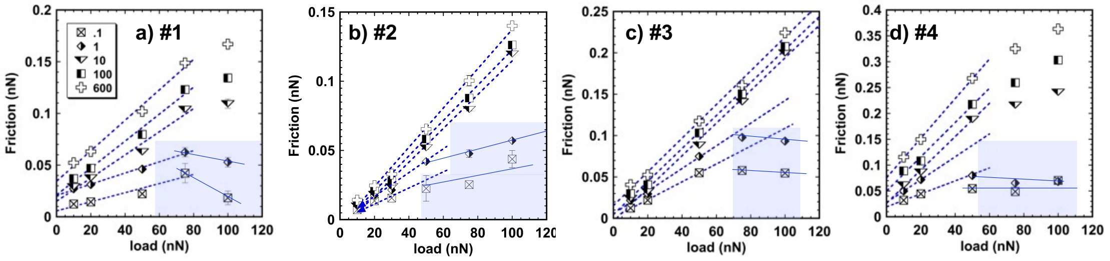

Figure S10. Friction as a function of load in aqueous solution at the selected velocities of 0.1, 1, 10, 100 and 600 μm/s measured for a) #1, b) #2, c) #3 and d) #4 calcite surface, respectively. The blue shadow indicates the change of slope (friction coefficient) due to pressure solution. The coefficient of friction μ_{PS} shown in Figure 7a was determined in the linear regime at loads ≥50 nN.

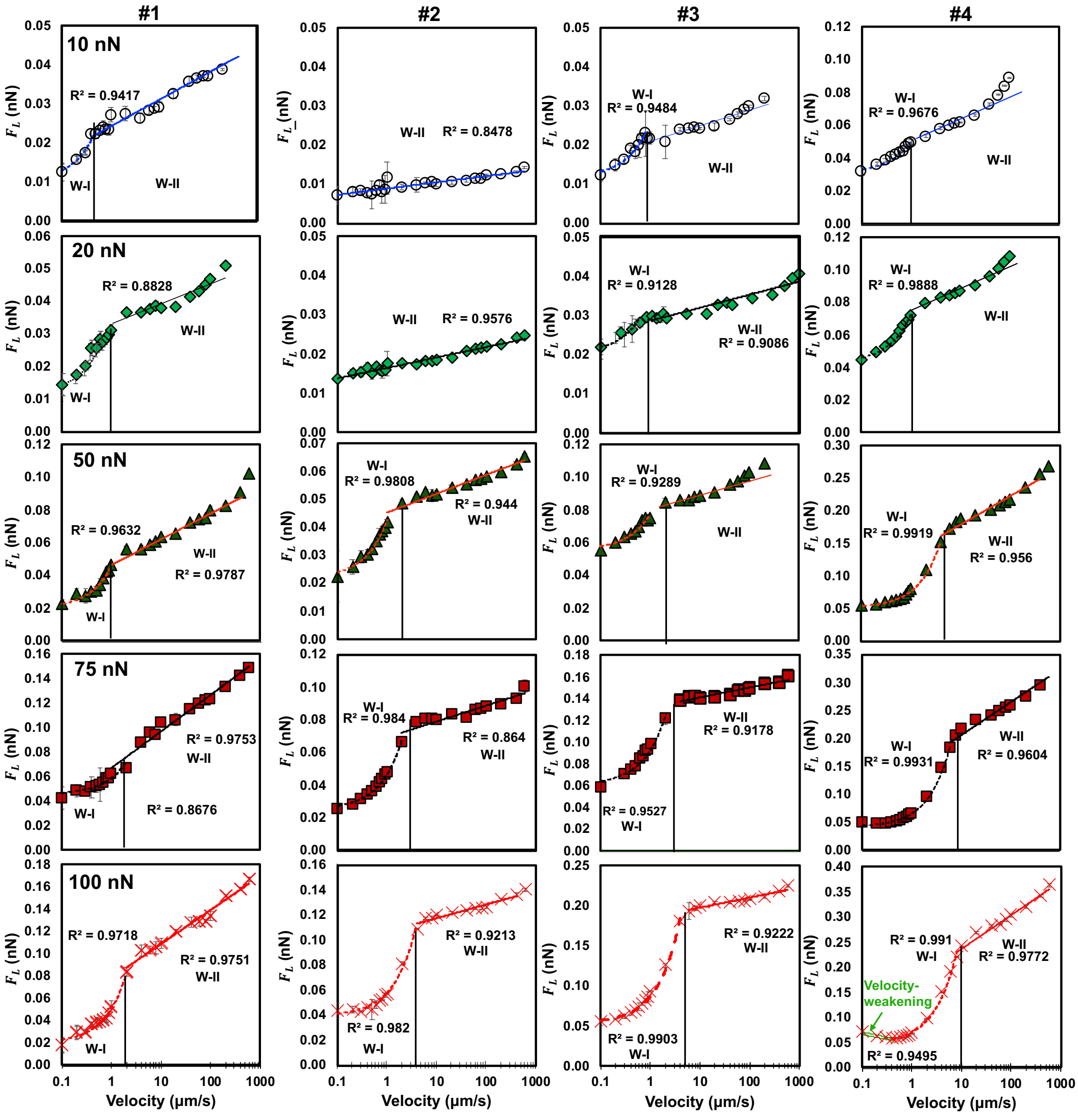

Figure S11. Friction force as a function of velocity in 1 mM $CaCl_2$ solution. Each plot represents the results at a single normal load for calcite #1 (column 1), #2 (column 2), #3 (column 3) and #4 (column 4) and 10 nN (first row), 20 nN (second row), 50 nN (third row), 75 nN (fourth row) and 100 nN (fifth row). The transition velocities V_{PS} and friction rate parameters α_{PS} and α_W are summarized in Table S3. The linear fit in W-I is shown by a dash line, while the fit to the Eyring model is given by a solid line. Note that the y axis is not same for each plot.

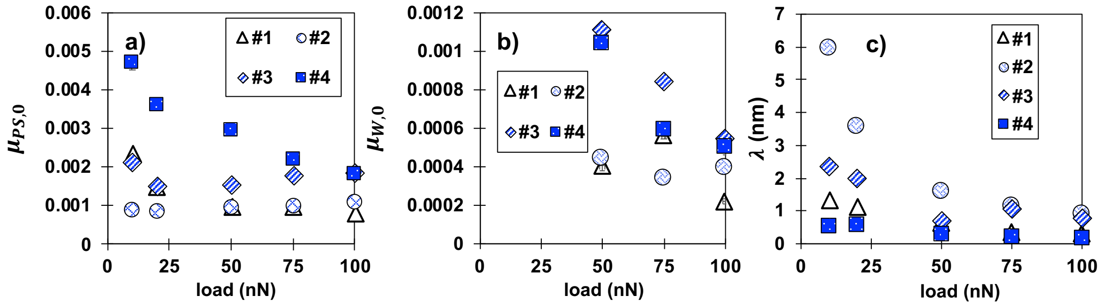

Figure S12. a) $\mu_{PS,0}$ as a function of normal load obtained from fitting $\mu_{W-I} = \mu_{PS,0} + \alpha_{PS}V$ to the experimental results in aqueous environment at $V < V_{PS}$ (W-I regime). b) $\mu_{W,0}$ as a function of normal load obtained from fitting $\mu_{W-II} = \mu_{W,0} + \alpha_W \ln V$ to the experimental results in aqueous environment at $V > V_{PS}$ (W-II regime). c) Correlation length λ as a function of load obtained from $\alpha_W = k_B T/(L\lambda)$. The decreasing trend of λ with load suggests that increasing confining pressure leads to thinner confined fluid films and thereby to smaller correlation lengths. The non-monotonic change of λ with RMS roughness is explain in the manuscript. The decreasing trend of $\mu_{PS,0}$ with load supports the pressure solution theory, as the increasing stress promoted calcite dissolution, and thereby, more efficient lubrication. The triangles are for #1 surface, circles for #2, diamonds for #3 and squares for #4.

Supplementary Tables

#calcite (RMS)		10 nN	20 nN	50 nN	75 nN	100 nN
**#1 (0.5 nm)**	**V***	40	40	40	40	40
	**β_D**	-0.00041	-0.000345	-0.000310	-0.000268	-0.000225
	**V****			200	200	200
	**α_D**			0.000110	0.000171	0.000290
**#2 (5 nm)**	**V***	1	1	10	10	10
	**β_D**	-0.00037	-0.0003385	-0.000156	-0.000116	-0.000059
	**V****	20	50	30	20	30
	**α_D**	0.000830	0.000500	0.00032	0.00032	0.000370
**#3 (10 nm)**	**V***					
	**β_D**					
	**V****			20	20	30
	**α_D**	0.00032	0.0001575	0.000262	0.000208	0.00024
**#4 (15 nm)**	**V***	0.7	1	1	0.7	0.7
	**β_D**	-0.0004	-0.00031	-0.000136	-0.0001627	-0.0000166
	**V****	3	3	2	2	1
	**α_D**	0.000288	0.00018	0.000144	0.00009985	0.00009467

Table S1. Fitting parameters for friction in dry environment: transition velocities from velocity weakening to plateau (V^* in µm/s) and from plateau to velocity strengthening (V^{**} in µm/s), slope in regime D-I (β_D) and in regime D-II (α_D). Error bars are shown in Figure 4.

	DMT model		Hertz model	
Load (nN)	a (nm)	stress (Gpa)	a (nm)	stress (Gpa)
10	5.59	0.61	3.03	0.35
20	5.87	0.64	3.82	0.44
50	6.59	0.67	5.18	0.59
75	7.08	0.75	5.93	0.68
100	7.52	0.81	6.53	0.75

Table S2. Contact radius a (nm) and average contact stress (GPa) for the contact between cleaved (atomically smooth) calcite surface and the silicon tip (R=190 nm) calculated using the DMT model ($\gamma = 0.044$ N/m) for adhesive contacts in dry environments and using the Hertz model for non-adhesive contacts in aqueous environment.

#calcite (RMS)		10 nN	20 nN	50 nN	75 nN	100 nN
#1 (0.5 nm)	$\mu_{PS,0}$	0.001378	0.000764	0.0004046	0.00056	0.00022
	α_{PS}	0.00132	0.000872	0.0005	0.00025592	0.00030594
	V_{PS}	0.4	1	1.5	2	2
	$\mu_{W,0}$	0.00232	0.00149	0.000946	0.000968	0.00078
	λ	1.33	1.09	0.61	0.36	0.31
	α_W	0.00031	0.0001885	0.0001348	0.00015387	0.000132
#2 (5 nm)	$\mu_{PS,0}$			0.00044	0.0003356	0.00039
	α_{PS}			0.00041594	0.00028667	0.00018
	V_{PS}			2	2.5	4
	$\mu_{W,0}$	0.00088	0.0008225	0.000932	0.00096	0.001081
	λ	5.96	3.58	1.57	1.15	0.90
	α_W	0.000069	0.000058	0.000053	0.000048	0.000046
#3 (10 nm)	$\mu_{PS,0}$	0.001015	0.00103	0.001111	0.00084	0.000546
	α_{PS}	0.00219	0.0010	0.000434	0.00041333	0.000325
	V_{PS}	0.8	0.8	2	3	5
	$\mu_{W,0}$	0.00211	0.001485	0.00151	0.00176	0.0018477
	λ	2.35	1.97	0.68	1.06	0.75
	α_W	0.0001750	0.0001044	0.0001211	0.0000517	0.0000547
#4 (15 nm)	$\mu_{PS,0}$	0.003234	0.00215	0.00104	0.000592	0.000503
	α_{PS}	0.00184	0.001505	0.0005	0.00029027	0.0002083
	V_{PS}	1	1	5	9	10
	$\mu_{W,0}$	0.00468	0.00358	0.00294	0.002176	0.001788
	λ	0.51	0.57	0.26	0.19	0.15
	α_W	0.000808	0.000359	0.000314	0.000284	0.000276

Table S3. Fitting parameters for friction in aqueous environment: transition velocity from linear (W-I) to logarithmic (W-II) regime (V_{PS} **in μm/s**); $\mu_{PS,0}$ (-) and α_{PS} (s/μm) are the intercept and slope in regime W-I ($\mu_{PS} = \mu_{PS,0} + \alpha_{PS}V$), respectively, and $\mu_{W,0}$ and α_W give the intercept and logarithmic slope in regime W-II ($\mu_W = \mu_{W,0} + \alpha_W \ln(V)$), and $\alpha_W = \frac{kT}{\lambda L}$, λ being the correlation length in nm. Error bars are shown in Figure 4 and S10.

References

1. Ruths M & Israelachvili JN (2011) Surface forces and nanorheology of molecularly thin films. *Nanotribology and Nanomechanics II*, (Springer), pp 107-202.
